# Efficient Ground-State Recovery of UV-Photoexcited *p*-Nitrophenol in Aqueous Solution by Direct and Multistep Pathways

**DOI:** 10.1021/jacs.4c10965

**Published:** 2024-10-25

**Authors:** Deborin Ghosh, K. Eryn Spinlove, Hallam J. M. Greene, Nicholas Lau, Sandra Gómez, Min-Hsien Kao, William Whitaker, Ian P. Clark, Partha Malakar, Graham A. Worth, Thomas A. A. Oliver, Helen H. Fielding, Andrew J. Orr-Ewing

**Affiliations:** † School of Chemistry, University of Bristol, Cantock’s Close, Bristol BS8 1TS, U.K.; ‡ Department of Chemistry, University College London, 20 Gordon Street, London WC1H 0AJ, U.K.; § Departamento de Química Física, Universidad de Salamanca, Salamanca, 37008, Spain; ∥ Central Laser Facility, Research Complex at Harwell, Science and Technology Facilities Council, Rutherford Appleton Laboratory, Harwell Oxford, Didcot, Oxfordshire OX11 0QX, U.K.

## Abstract

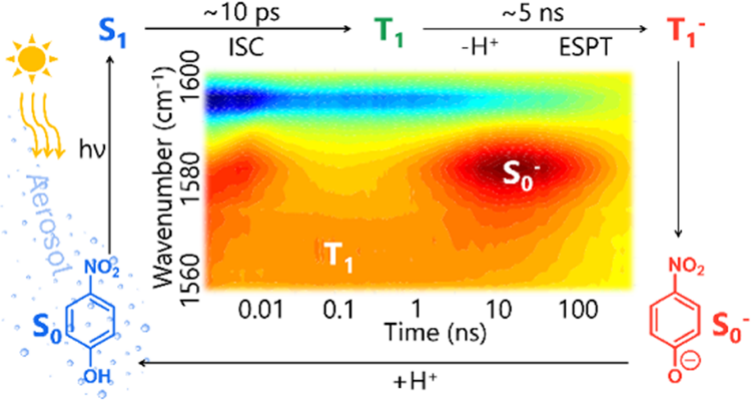

Nitroaromatic compounds are found in brown carbon aerosols emitted to the Earth’s atmosphere by biomass burning, and are important organic chromophores for the absorption of solar radiation. Here, transient absorption spectroscopy spanning 100 fs–8 μs is used to explore the pH-dependent photochemical pathways for aqueous solutions of *p*-nitrophenol, chosen as a representative nitroaromatic compound. Broadband ultrafast UV–visible and infrared probes are used to characterize the excited states and intermediate species involved in the multistep photochemistry, and to determine their lifetimes under different pH conditions. The assignment of absorption bands, and the dynamical interpretation of our experimental measurements are supported by computational calculations. After 320 nm photoexcitation to the first bright state, which has ^1^ππ* character in the Franck–Condon region, and ultrafast (∼200 fs) structural relaxation in the adiabatic S_1_ state to a region with ^1^nπ* electronic character, the S_1_*p*-nitrophenol population decays on a time scale of ∼12 ps. This decay involves competition between direct internal conversion to the S_0_ state (∼40%) and rapid intersystem crossing to the triplet manifold (∼60%). Population in the T_1_-state decays by excited-state proton transfer (ESPT) to the surrounding water and relaxation of the resulting triplet-state *p*-nitrophenolate anion to its S_0_ electronic ground state in ∼5 ns. Reprotonation of the S_0_-state *p*-nitrophenolate anion recovers *p*-nitrophenol in its electronic ground state. Overall recovery of the S_0_ state of aqueous *p*-nitrophenol via these competing pathways is close to 100% efficient. The experimental observations help to explain why nitroaromatic compounds such as *p*-nitrophenol resist photo-oxidative degradation in the environment.

## Introduction

Brown carbon (BrC) aerosols are released into the Earth’s atmosphere by biomass burning, from wildfires, deforestation, or agricultural practices, and from residential heating by wood or coal combustion.^1^ They also form through photochemical processing of organic compounds present in the atmosphere as gaseous constituents, dispersed particulates, or dissolved in aqueous microdroplets. These BrC particles absorb solar visible and ultraviolet wavelengths, trapping the energy from this solar radiation as well as promoting evaporation of water from cloud droplets, hence they contribute both directly and indirectly to atmospheric radiative forcing.^2^ BrC aerosol particles contain a variety of organic compounds, and are distinct from soot-like black carbon particles in their optical and chemical properties. Whereas black carbon particles absorb strongly across the UV, visible and IR regions, the molecular composition of BrC particles gives spectra characterized by increasing absorption from the visible to the UV.^1^ Analysis of the light-absorbing chromophores in BrC particles sampled from the troposphere has shown that a significant fraction of the absorption at wavelengths longer than 300 nm is by nitroaromatic compounds.^3^ These nitroaromatic molecules are produced either in the gas or particulate phases by OH or NO_3_ radical initiated oxidation of aromatic species, including toluene and phenolic compounds such as catechols, in the presence of NO_*x*_.^4−7^

Example nitroaromatic compounds identified in BrC aerosol sampled from the atmosphere include nitrophenols, nitrocatechols, and nitromethoxyphenols.^3^ In atmospheric BrC aerosols, the nitroaromatic compounds will be dispersed in media comprising other organic constituents and water taken up from the surrounding air, with the water fraction dependent on the ambient relative humidity. Their photodegradation rates are affected by the properties of the surrounding matrix, such as its water or organic content and the viscosity.^8−10^ For nitrophenols and nitrocatechols, the absorption spectra of the phenolic and deprotonated phenolate anion forms differ, as do their photochemical pathways, and hence the photochemical processing of these compounds in the atmosphere will depend on both the p*K*_a_ values of the nitroaromatic species and the pH of their aerosol environments.^7^ These and other nitroaromatic compounds such as 2,4-dinitrophenol are also present in effluent streams from a variety of industrial processes, and are known to be persistent and toxic environmental pollutants that are resistant to biological and UV photochemical degradation.^10−12^

Under solar irradiation in the troposphere, and in the presence of atmospheric oxidizers such as OH radicals, ozone, and O_2_, photochemical processing of the organic constituents of BrC will change their chemical composition over time. Although excited triplet states of nitroaromatic compounds are thought to react with other organic compounds by hydrogen atom abstraction,^13,14^ and hydrolysis in aqueous solution,^11^ quantum yields for photochemical degradation of nitrophenols are low in both organic and aqueous media. From their studies of such quantum yields for various nitrophenols in 2-propanol and water solutions, Dalton et al. estimated atmospheric lifetimes to solar degradation of a few days or longer.^15^

Here, we explore the photochemical fates of a model nitroaromatic chromophore, *p*-nitrophenol (*p*-NP), in aqueous solution, and the dependence of its photochemistry on pH. The longest wavelength bands in the UV–visible absorption spectra of aqueous *p*-NP solutions shift further into the visible region at pH ≥ 7 because of deprotonation of the −OH group. Of significance to the tropospheric photochemistry of *p*-NP, it exhibits absorption bands assigned to π* ← π orbital excitations^16^ that extend to wavelengths longer than 290 nm, and hence overlap with the solar flux transmitted through the stratosphere.

The gas-phase photochemistry of various phenol derivatives has been studied extensively both computationally and experimentally, for example by investigation of the H atom loss channel resulting from crossings between excited ^1^ππ* states and states with repulsive ^1^πσ* character.^17−20^ Substitution at the 4-position, para to the −OH group with, for example, an −NO_2_ group, affects the S_0_ – S_1_ excitation energies, O–H bond dissociation energies, and ionization energies of the phenol derivatives in ways that can be modeled quantitatively by Hammett-like parameters for the substituent groups.^21^ However, detailed studies of the photochemical dynamics of gas-phase nitrophenols are sparse. Domcke and co-workers argued that in aqueous solution, the πσ* states in phenol and other heteroaromatic compounds become states with charge-transfer to solvent character,^19,20^ resulting in autoionization pathways for UV-excited phenol.^22^ A prior study of *p*-nitrophenol photochemistry at 266 nm in aqueous solution assigned an observed transient absorption (TA) band centered near 700 nm to production of solvated electrons,^12^ whereas Unterreiner and co-workers identified the formation of *p*-nitrophenolate anions over time scales of a few nanoseconds.^23^ Ultrafast transient absorption spectroscopy measurements of 400 nm photoexcited ortho, meta and para-nitrophenolate anions in basic aqueous solution by Michenfelder et al. and by Bailey-Darland et al. showed loss of stimulated emission (SE) and excited state absorption (ESA) bands within a few hundred fs (for *o*- and *p*-nitrophenolate, but with longer time scales for *m*-nitrophenolate) because of rapid relaxation to the ground electron state through an S_1_ – S_0_ conical intersection accessed by torsional motion of the −NO_2_ group on the S_1_-state potential energy surface (PES).^24,25^ The photochemical dynamics of various other nitroaromatic compounds such as nitrobenzene,^26−33^*o*-nitrotoluene,^34^ 1-nitronaphthalene,^35−37^ 2-nitronaphthalene,^37,38^ 1-nitropyrene,^39^ nitrobenzaldehydes,^40^*p*-nitroaniline,^41^ and larger derivatives,^42,43^ have also been explored in solution and in the gas phase using experimental and computational methods. In many cases, the nitroaromatic chromophore in these molecules promotes intersystem crossing (ISC) to the triplet manifold of excited states on femtosecond to picosecond time scales.^43^ Substitution of the aromatic ring ortho to the −NO_2_ group can facilitate intramolecular isomerization or reaction,^16,44,45^ such as excited state intramolecular proton transfer (ESIPT) and the reported elimination of HONO from *o*-nitrophenol in the gas phase.^46,47^

Here, we use transient absorption spectroscopy on time scales spanning 100 fs–8 μs, and with spectroscopic observation in both the UV–visible and mid-IR regions, to track the multistep photochemical pathways that follow 320 nm UV excitation of aqueous solutions of *p*-nitrophenol and 400 nm photoexcitation of aqueous *p*-nitrophenolate. The 320 nm radiation excites the longest wavelength absorption band of *p*-nitrophenol to initiate photochemistry analogous to that for aqueous aerosol droplets in the Earth’s troposphere exposed to solar flux at wavelengths longer than 290 nm. Our spectroscopic detection identifies several excited states and reactive intermediates playing significant roles in the photochemistry, and it quantifies the recovery of the parent *p*-nitrophenol by various competing relaxation pathways. Because *p*-NP is a weak acid (p*K*_a_ = 7.2), the pH dependence of the photochemistry is also explored. The outcomes demonstrate the resistance of aqueous *p*-nitrophenol to photochemical degradation, and they contribute to an understanding of the photochemical processing of brown carbon aerosol, as well as the persistence of polluting nitroaromatic compounds in the environment.

## Results and Discussion

### Steady-State Spectroscopy

**Figure 1:**
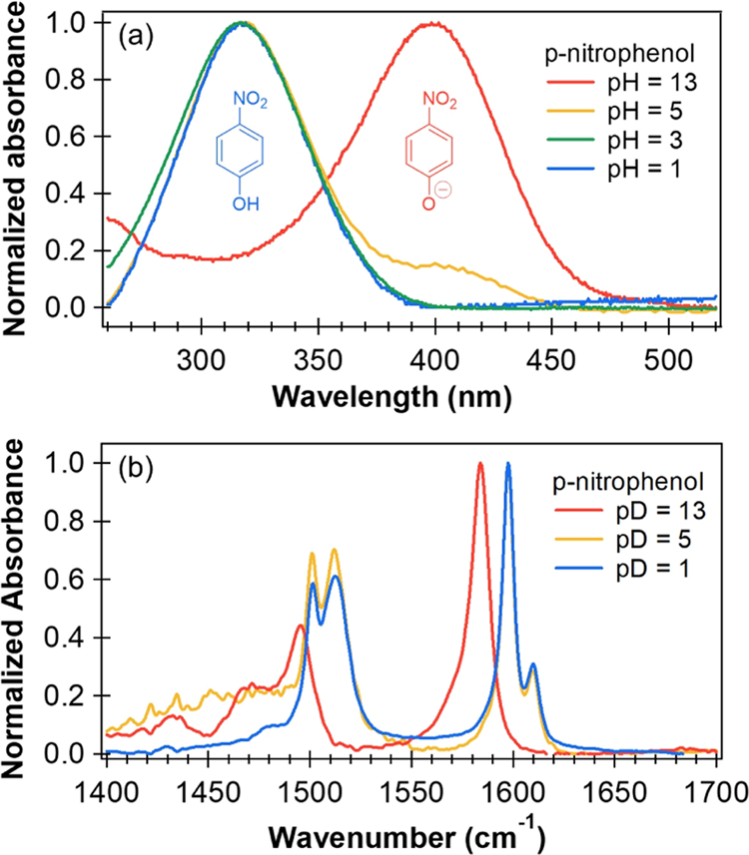
(a) Steady-state UV–vis absorption spectra and (b) background subtracted Fourier transform infrared (FTIR) spectra of *p*-NP (p*K*_a_ = 7.2) in aqueous solutions of various pH/pD values. The FTIR spectra were recorded for solutions in D_2_O. The structures of the protonated (blue) and deprotonated (red) forms of *p*-NP are included in (a), positioned under the UV–vis absorption bands for which they are responsible.

Steady-state UV–visible absorption spectroscopy provides an initial characterization of the electronically excited states involved in the photochemistry of aqueous *p*-nitrophenol and *p*-nitrophenolate. The orbital excitations associated with the near-UV and visible absorption bands for *p*-NP and *p*-nitrophenolate plotted in Figure 1a are shown in Figure S1 of Supporting Information, together with predicted wavelengths. These absorption bands arise from strong π* ← π, LUMO ← HOMO excitations. The calculations successfully capture the shift of the absorption band to longer wavelength with deprotonation of the phenol group. The first few calculated excited electronic energy levels in the Franck–Condon regions of *p*-NP and *p*-nitrophenolate, microsolvated by three water molecules, along with their characters, transition energies, and oscillator strengths, are listed in Table S1 of the Supporting Information. Plots of the molecular orbitals involved in the transitions listed in Table S1 are shown in Figure S2 of the Supporting Information. Attempts to measure fluorescence emission spectra from *p*-nitrophenol in solutions in water were unsuccessful, suggesting that the S_1_ excited electronic state has a short lifetime compared to the time scale for radiative decay, and hence a low fluorescence quantum yield.

The absorption spectra guided the choice of excitation wavelengths of 320 nm for *p*-NP and 400 nm for *p*-nitrophenolate in our transient absorption spectroscopy experiments. In addition, further measurements were made for *p*-NP at 310 and 360 nm excitation wavelengths which are reported in the Supporting Information (SI).

Steady-state FTIR spectra for aqueous *p*-NP (see Figure 1b) show absorption bands in the 1400–1700 cm^–1^ mid-IR window centered at 1501, 1512, 1597, and 1610 cm^–1^. In basic solution, the corresponding IR absorption bands for deprotonated *p*-nitrophenolate shift to lower wavenumber (1470, 1495, and 1584 cm^–1^), allowing the neutral and anionic species to be distinguished. According to previously published density functional theory (DFT) calculations,^48^ the IR absorption bands around 1600 cm^–1^ are primarily attributed to the C–C bond vibrations of the aromatic ring, while the bands around 1500 cm^–1^ mainly result from the asymmetric stretching of the −NO_2_ group and in-plane C–H bending.

### Transient Absorption Spectroscopy

**Figure 2:**
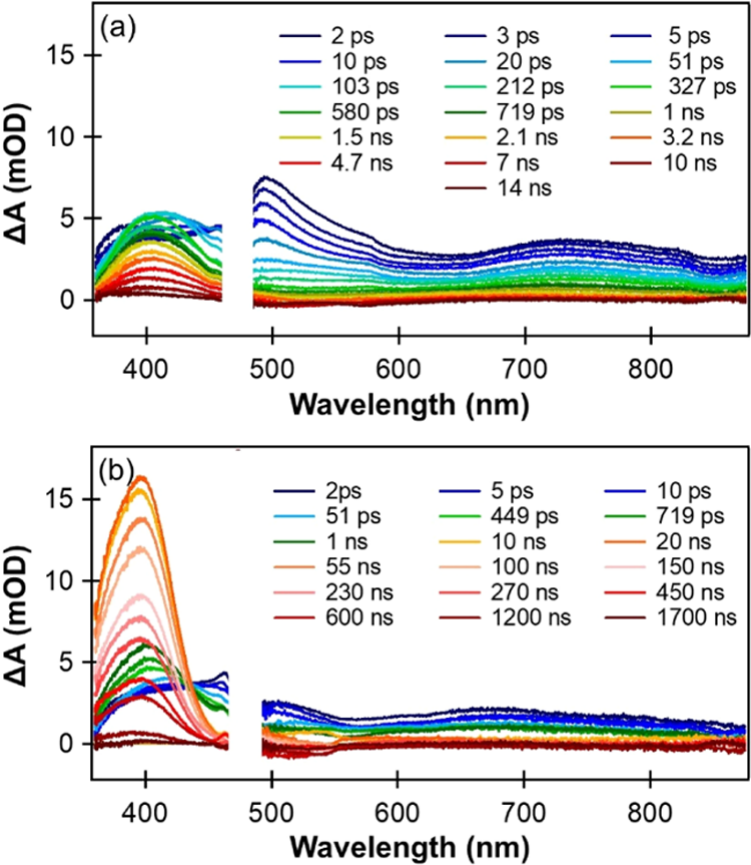
TA spectra (recorded at RAL) of aqueous *p*-NP at different time delays for (a) pH 1 and (b) pH 5 solutions. In all the measurements, 320 nm UV pump light was used for photoexcitation. The inset keys show the colors used to plot spectra measured at different pump–probe time delays. The white box in each panel masks the region between white-light continuum (WLC) probe ranges where the probe light intensity is insufficient for measurements.

**Figure 3:**
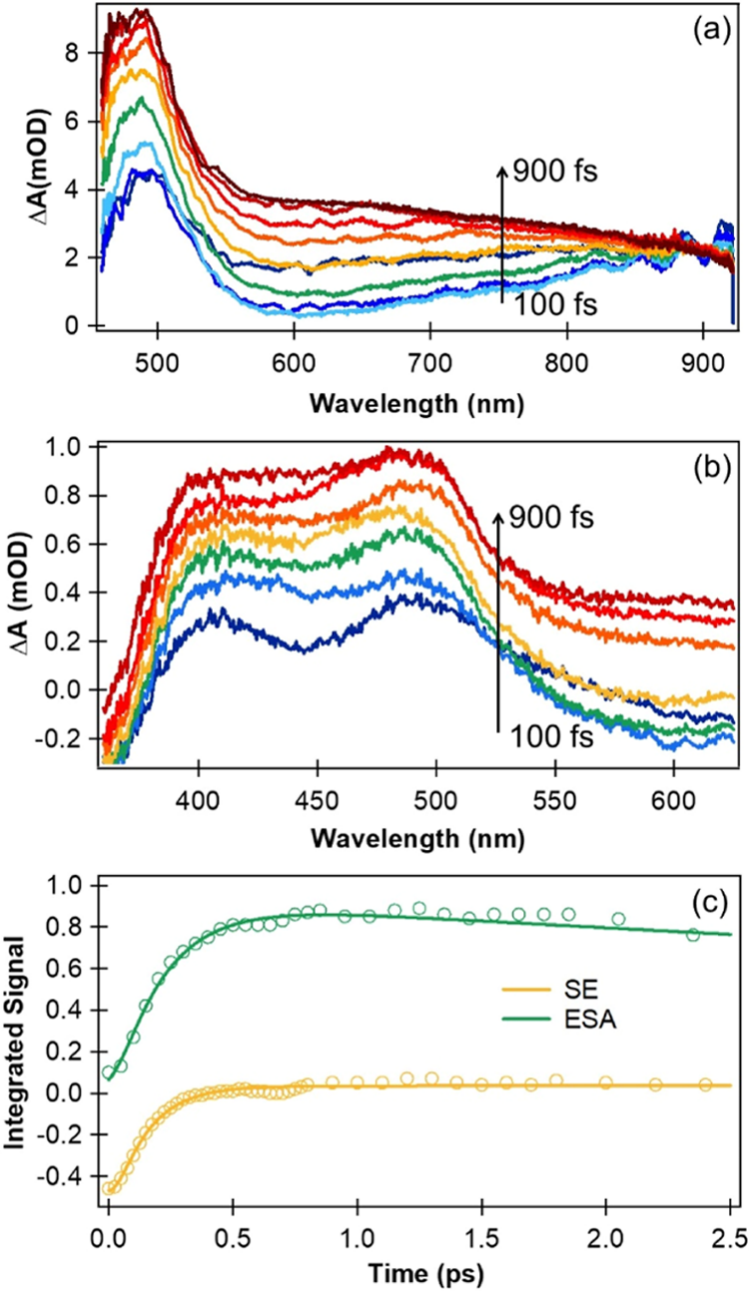
Early time (<1 ps) TA spectra of aqueous *p*-NP (pH 5) obtained at (a) RAL and (b) UoB after photoexcitation at 320 nm. The two experimental systems provide complementary probe wavelength ranges. These TA spectra contain contributions from an SE band centered around 600 nm and a dual ESA band with peaks around 380 and 500 nm. (c) Kinetics of the SE decay and the ESA bands at time delays up to 2.5 ps, derived from the time-dependent integrated band intensities after spectral decomposition using KOALA software. Solid lines are fits to mono or biexponential functions convoluted with the experimental IRF.

**Figure 4:**
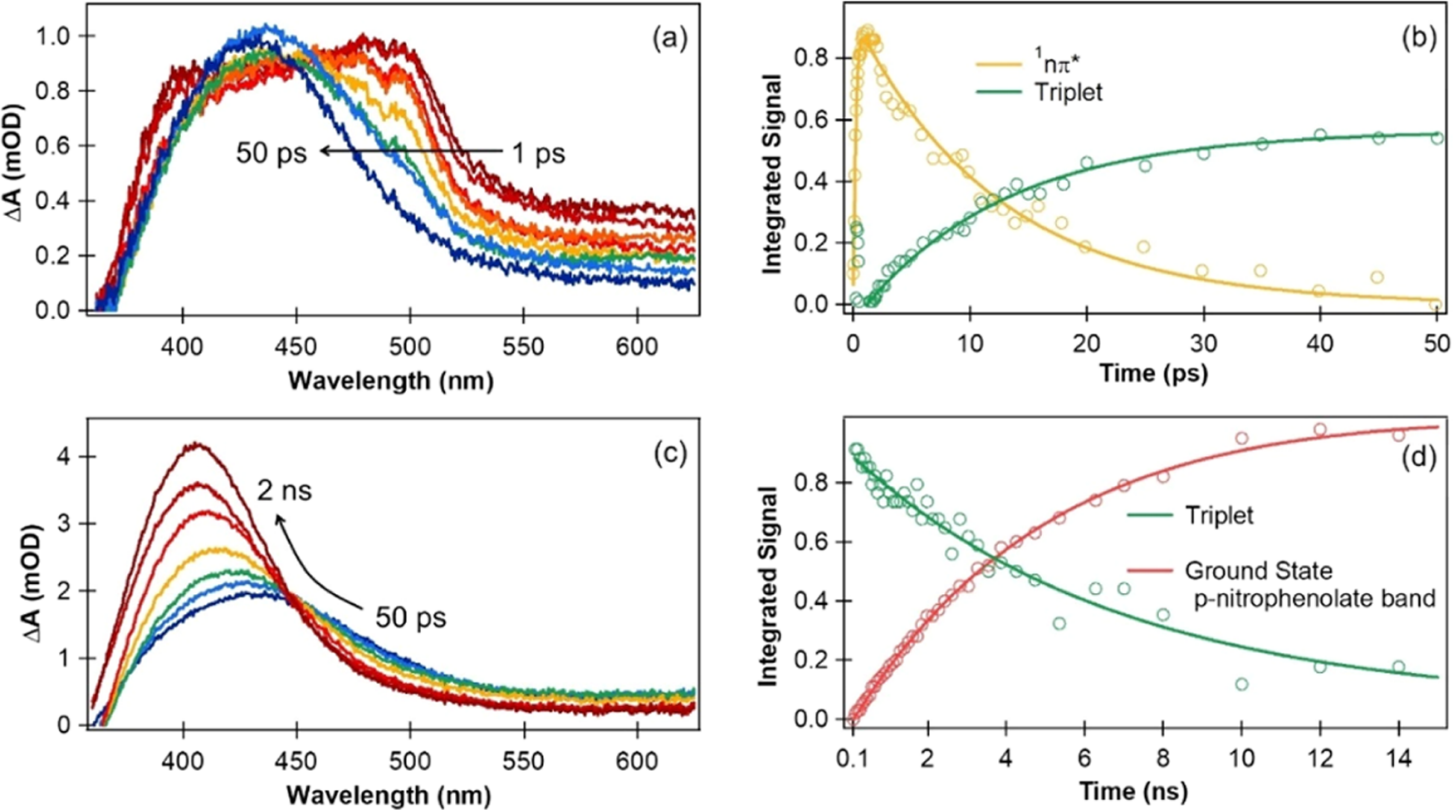
TA spectra for aqueous *p*-NP (pH 5) photoexcited at 320 nm recorded at time delays up to 2 ns, and derived kinetic traces following spectral decomposition using KOALA. (a) TA spectra from 1–50 ps recorded at UoB and (b) kinetics of *p*-NP, for which growth of triplet-state population by ISC from the S_1_(nπ*) state is observed. (c) TA spectra from 50 ps–2 ns recorded at UCL and (d) kinetics of *p*-NP in which the ground state *p*-nitrophenolate band intensity grows as the triplet state ESA decays.

Example TA spectra of 2 mM aqueous solutions of *p*-NP in deionized water (giving a pH 5 solution because of the weak acidity of *p*-NP) and with added HCl (to obtain pH 1) are shown in Figure 2, while those at pH 3 are presented in Figure S3 of the Supporting Information. Under all these pH conditions, the degree of dissociation of *p*-NP is small (e.g., 0.006 for the 2 mM aqueous solution of *p*-NP in deionized water without added HCl), so any contributions from the photochemistry of the deprotonated *p*-NP are negligible. These TA measurements were made using the LIFEtime instrument at the Rutherford Appleton Laboratory (RAL), for which delay times extend from <1 ps to beyond 1 μs. Several distinct spectroscopic features are observed and are highlighted in expanded spectra obtained over narrower time delay windows plotted in Figures 3–4. These spectra were measured using the RAL, University of Bristol (UoB), and University College London (UCL) TA systems. At subpicosecond time delays, Figure 3a and the spectral decomposition in Figure S4 of Supporting Information show that the TA spectra are dominated by a stimulated emission (SE) band centered around 600 nm, which disappears within 1 ps. The TA spectra then evolve into excited-state absorption (ESA) bands centered near 380 nm, 500 nm, and 700–800 nm (Figures 2 and 3b). These bands decay with increasing time delay, first forming a broad feature spanning 400–500 nm (Figure 4a) in solutions at all pHs, and later transitioning to a sharper feature peaking at 400 nm in pH 3 and pH 5 solutions (Figures 2b and 4c). At pH 3 and pH 5, this latter feature matches the known steady-state absorption spectrum of the *p*-nitrophenolate anion, recorded by UV–visible spectroscopy of an aqueous solution of *p*-NP with added sodium hydroxide (see Figure S5 of Supporting Information). The interpretation of the other spectral features is discussed by analogy to prior experimental studies of the UV photochemistry of aqueous phenol^22^ and aqueous nitrobenzene.^30^

The SE band peaking at 600 nm, and dual ESA band peaking near 380 and 500 nm, are observed in the earliest time spectra. Because these bands overlap, the combined change in absorbance, Δ*A*, remains positive at all probe wavelengths, but spectral decomposition illustrated in Figure S4a of Supporting Information shows that the SE contribution to Δ*A* is negative, as expected. Experiments conducted with a 200 fs instrument response (IRF) at RAL and a 110 fs instrument response at UoB,^49^ examples of which are shown in Figure 3a,b, reveal a rapid loss (τ_SE_ ≤ 200 fs) of SE and a similarly rapid rise with a time constant of evolution (τ_evo_ = 201 ± 22 fs) of the dual ESA band. The kinetics of the integrated band intensities are shown in Figure 3c, together with fits to exponential functions convoluted with the experimental IRF. The fast decay of the SE and growth of the ESA are consistent with our quantum dynamical calculations for *p*-NP microsolvated by three water molecules which show transfer of population from the initially photoexcited ^1^ππ* region of the adiabatic S_1_ state to a dark ^1^nπ*-character region of S_1_ (see Figure S6 of the Supporting Information). The 600 nm feature in our TA spectra is therefore assigned to SE from the adiabatic S_1_ state, which has ^1^ππ* character in the Franck–Condon region, but this SE is lost as the S_1_ state evolves to the region of ^1^nπ* character after structural relaxation in the excited state. Hence, the dual ESA band is assigned to transient absorption from the S_1_(nπ*) state. The two prompt ESA bands decay in intensity with an exponential time constant of 12.2 ± 0.4 ps, and we see commensurate growth of the broad 400–500 nm feature (see Figure 4a,b), which resembles a feature seen in TA spectroscopy of aqueous nitrobenzene that was assigned to ESA from the T_1_ state,^30^ albeit without the resolved vibronic structure observed for photoexcited nitrobenzene. Our quantum chemical calculations for *p*-NP support rapid population of a triplet state from the S_1_(nπ*) state: as is shown in Figure S7, the S_1_(nπ*) state and T_2_(ππ*) state are energetically close at the minimum energy geometry of the S_1_(nπ*) state and, importantly, the calculated spin orbit coupling (SOC) between these two states is significant (41.2 cm^–1^). Hence, we assign this 400–500 nm ESA band to population of the T_1_ state of *p*-NP. Zobel et al. observed a similar ultrafast evolution of singlet excited state character, followed by ISC, in their *ab initio* nonadiabatic molecular dynamics studies of 1-nitronaphthalene, 2-methyl-1-nitronaphthalene, and 2-nitronaphthalene.^37,38^ An alternative assignment to the *p*-nitrophenoxyl radical is discussed briefly and discounted below. The growth of the 400 nm band assigned to ground-state *p*-nitrophenolate occurs on a 5 ns time scale, independent of solution pH (for pH 3 to 5) and is accompanied by decay of the 400–500 nm broad ESA feature (see Figure 4c,d).

The broad TA absorption band centered around 700–800 nm (Figure 2) merits careful consideration. It is reminiscent of the absorption spectrum of aqueous solvated electrons (*e*_aq_^–^),^50−52^ with the shorter wavelength wing masked by the overlapping ESA bands discussed above. A similar band was seen in nanosecond TA experiments by Zhao et al. following 266 nm photoexcitation of aqueous *p*-NP, and these authors attributed it to solvated electrons with support from quenching studies using dissolved N_2_O.^12^ However, various considerations argue against assignment of this feature to a solvated electron at the longer excitation wavelengths used in our work, and we instead adopt an alternative assignment to *p*-NP S_1_ and T_1_ ESA bands. The latter assignment is reasonable given the known propensity for numerous nitroaromatic compounds with low-lying excited nπ* states to relax to the ground state through their triplet states.^27,35,36,39^ Our assignment is also supported by observation of a similar feature in TA spectra of aqueous nitrobenzene,^30^ and for experiments conducted on *p*-NP in MeCN and chloroform solutions (see Section S2 and Figure S8b,d of the Supporting Information). Thermodynamic considerations described in Section S3 (Figure S9 and Tables S2 and S3) in the Supporting Information argue against production of a solvated electron from 320 nm single-photon excitation of *p*-NP. Although autoionization was reported for aqueous phenol after 267 nm photoexcitation,^22^ the few-nanosecond time scale observed for electron tunneling is much longer than the kinetics we see for growth of the 700–800 nm band for *p*-nitrophenol. Our further test experiments at wavelengths of 310 and 360 nm, in which the same feature is also observed on similar time scales (see Figure S9), provide further evidence in support of our preferred assignment and against a tunneling autoionization mechanism. We also note that the kinetics of decay of this feature (and their dependence on pH) are better explained by an S_1_/T_1_ ESA assignment, as the two decay time constants for this band (Table S3) are in good agreement with the lifetimes of the ^1^nπ* state and the triplet state determined from other ESA features. This analysis indicates that the broad band around 700–800 nm has overlapping features from the ESA of the ^1^nπ* state and the triplet state, but the low intensity and breadth of the band make it difficult to distinguish the individual absorption contributions.

Ruling out an autoionization pathway from the *p*-nitrophenol S_1_(nπ) state allows us to reject any formation of the *p*-nitrophenoxyl radical by rapid H^+^ elimination from the radical cation of *p*-nitrophenol, again in contrast to the analogous photochemical pathway identified for phenol photoexcited at shorter UV wavelengths in aqueous solution.^22^ The *p*-nitrophenoxyl radical can therefore be discounted as responsible for any of the observed 400–500 nm bands in our TA spectra. We also note that the 5 ns growth of the distinct *p*-nitrophenolate band at 400 nm is too fast for diffusive recombination of a solvated electron and a *p*-nitrophenoxyl radical, which would require hundreds of nanoseconds or longer under our experimental conditions. An alternative route to the *p*-nitrophenolate anion must therefore be identified.

### TRIR Spectroscopy

**Figure 5:**
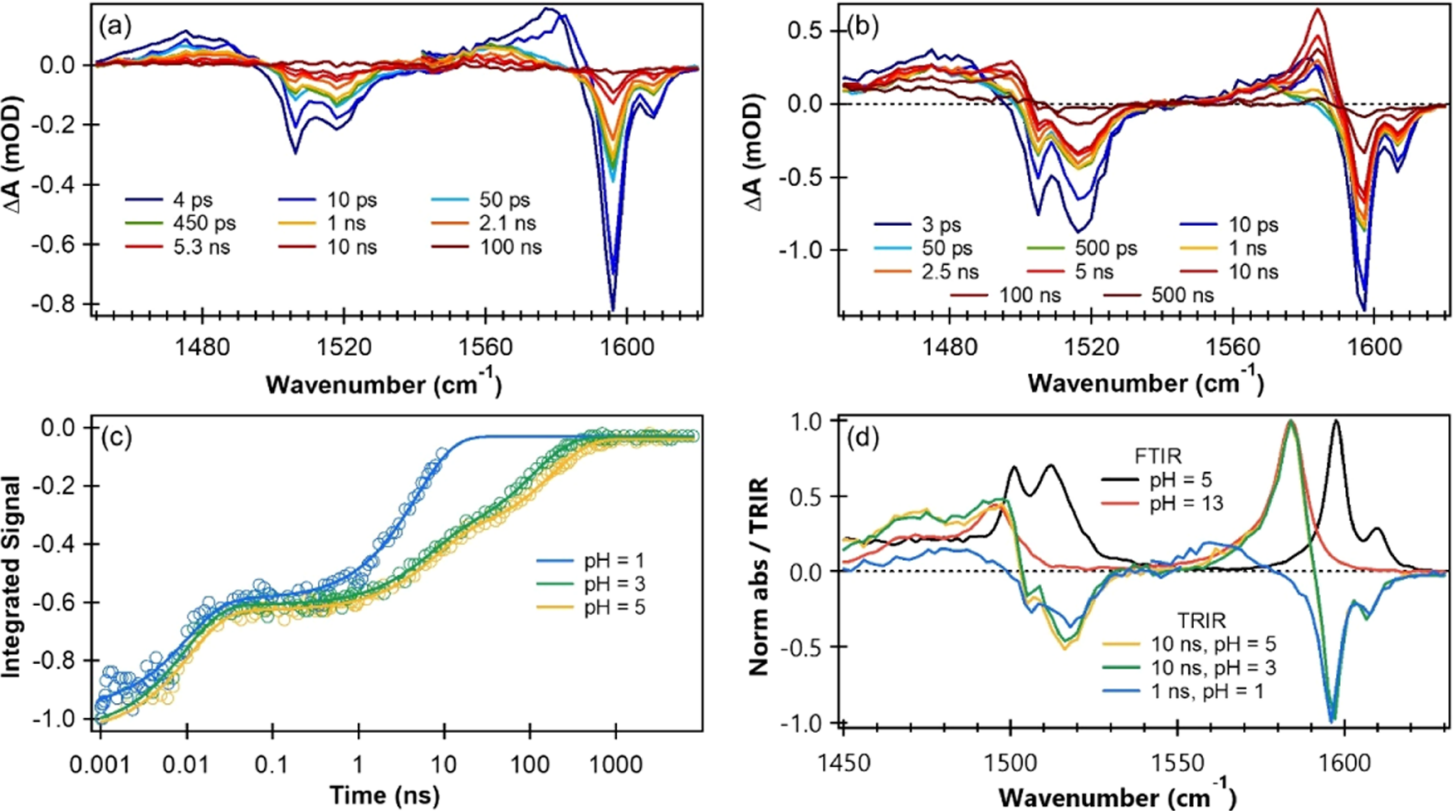
Photodynamics of aqueous *p*-NP excited at 320 nm and observed over picosecond to microsecond time delays by TRIR spectroscopy. Selected TRIR spectra are shown for (a) pD 1 and (b) pD 5 solutions in D_2_O. (c) Kinetics of *p*-NP (S_0_) recovery after photoexcitation of *p*-NP in pD 1, 3, and 5 solutions, obtained from the time dependence of the integrated intensities of the 1597 cm^–1^ GSB feature. (d) Normalized TRIR spectra of aqueous *p*-NP at a time delay of 1 ns in pD 1 solution, and at 10 ns in pD 3 and 5 solutions. Normalized FTIR spectra of *p*-NP and *p*-nitrophenolate (black and red solid lines, respectively) are included for comparison.

Time-resolved infrared (TRIR) spectra were obtained for 5 mM *p*-NP solutions in D_2_O with control of pD by addition of aqueous (D_2_O) DCl solution. Representative TRIR spectra for 320 nm excitation of *p*-NP at pD 5 and at pD 1 are shown in Figure 5a,b, while those at pD 3 are presented in Figure S10 of the Supporting Information. Negative features match the band centers and shapes of absorption bands seen in the steady-state FTIR spectra, and therefore correspond to depletion of ground-state molecules by photoexcitation. Such features are referred to as ground-state bleaches (GSBs). Their recoveries with increasing pump–probe delay time reflect the relaxation pathways that return the photoexcited *p*-NP molecules back to *p*-NP (S_0_). Within the signal-to-noise levels of our measurements, these recoveries are 100% complete on the nanosecond to microsecond time scales of TRIR measurements with the RAL LIFEtime laser system. Plots of the time-dependence of the integrated band intensities of the GSB features at 1597 cm^–1^ are shown in Figure 5c and can be fitted to extract the exponential time constants for ground-state population recovery. This analysis reveals that at pD 5, the *p*-NP (S_0_) molecules recover on time scales of 10.2 ± 0.6 ps, 7.2 ± 0.5 ns, and 180 ± 10 ns (see Table S4 of the Supporting Information). The recovery time constants show a nearly identical trend at pD 3, with the major difference occurring in the slowest recovery channel, which is relatively faster (109 ± 7 ns) at pD 3. However, at pD 1, there are only two observed recovery channels, and the time constants are 10.1 ± 0.9 ps and 5.2 ± 0.2 ns. The relative amplitudes of the multiexponential fit components used to estimate the fractional recovery by each pathway are reported in Table S4.

Positive features evident in the TRIR spectra shown in Figures 5 and S10 correspond to either excited-state absorptions or IR signatures of intermediates or products of the photochemical reactions that follow 320 nm excitation. Comparison to the steady-state IR spectrum in Figure 5d allows bands in the TRIR spectra at 1584, 1495, and 1470 cm^–1^ to be assigned to *p*-nitrophenolate. These features show matching kinetics to the 400 nm band observed in TA spectra (see Figure S11), confirming this band assignment. Note that these *p*-nitrophenolate bands are not observed for pD 1 solutions because of rapid protonation of the anions on time scales faster than those for their formation. Further positive-going features observed on the low wavenumber side of the GSBs until 25 ps after photoexcitation are characteristic of anharmonically shifted IR absorptions caused by vibrationally excited, yet electronically ground state, *p*-NP molecules (see Figure S12a). These hot-ground-state absorption (HGSA) features are attributed to rapid and direct IC from the S_1_ state to the ground S_0_ state. The HGSA bands decay in a few ps (see Figure S12b) because of efficient vibrational energy transfer to the surrounding solvent (i.e., vibrational cooling of the internally hot S_0_ state *p*-NP). Further weak features in the TRIR spectra, apparent at 1475 and 1568 cm^–1^ grow and decay on time scales in accord with the broad feature observed at 400–500 nm in TA spectra, and are therefore assigned to the *p*-NP (T_1_) state (see Figure S13).

### Photochemical Mechanism

**Scheme 1:**
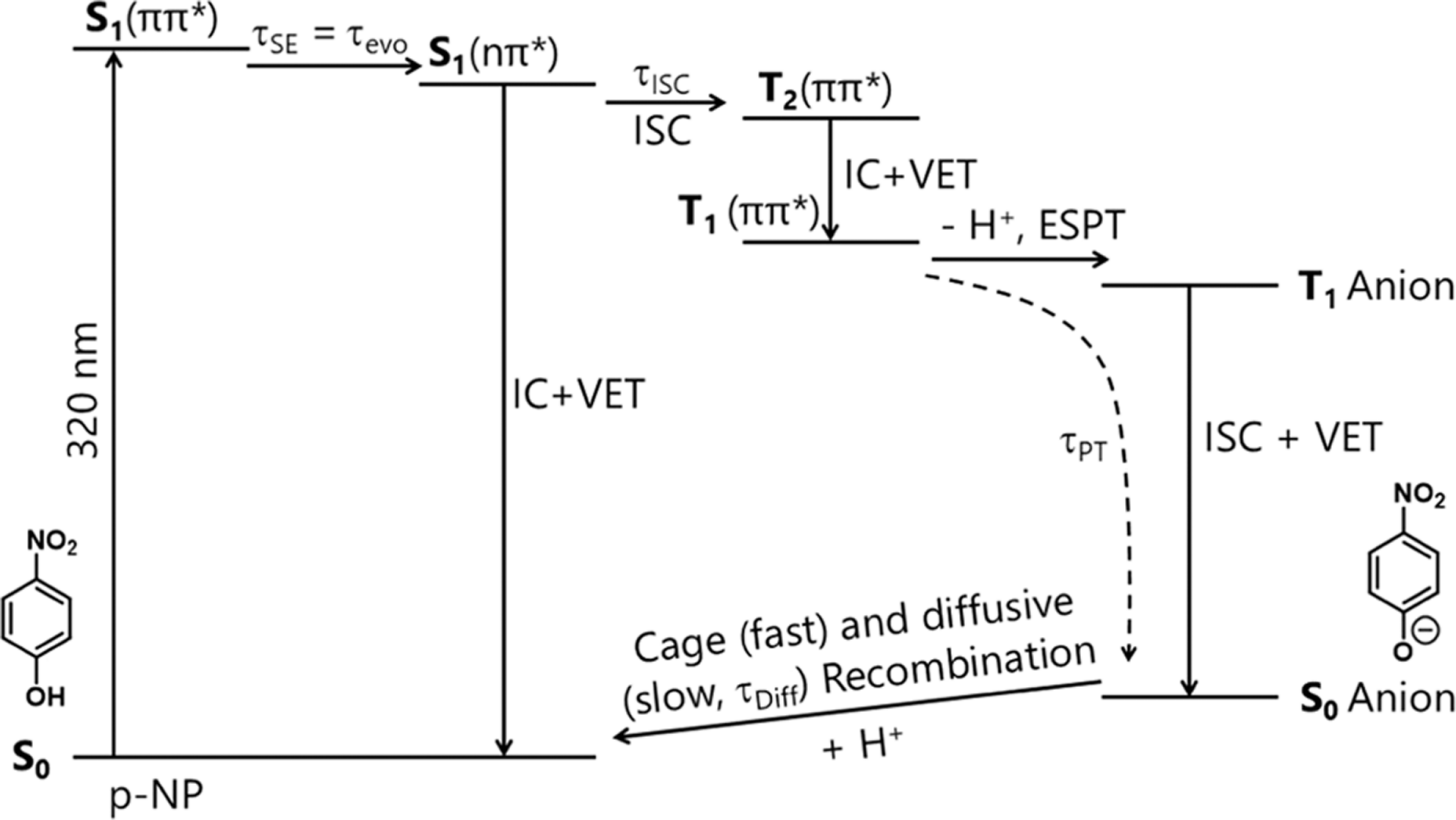
Photochemical Pathways for Relaxation of Aqueous *p*-NP after Photoexcitation at 320 nm Internal energy is conserved during IC and ISC processes but is lost through vibrational energy transfer (VET) to the surrounding solvent. Solid arrows represent photo-induced processes labeled by the time constants associated with those processes, as measured by our transient spectroscopy experiments. The dashed arrow represents the deprotonation time constant, which is associated with two consecutive steps.

Scheme 1 shows photochemical pathways and time constants (with values summarized in Table 1) for aqueous *p*-NP following photoexcitation at 320 nm that are consistent with all our TA and TRIR measurements over subpicosecond to microsecond time scales and under acidic conditions. Transient behaviors of the S_1_ and T_1_ states, and the ground state *p*-nitrophenolate are identified and distinguished through a combination of these TA and TRIR measurements, with support from electronic structure and quantum dynamics calculations. Although Scheme 1 attributes differently labeled time constants τ_evo_ and τ_SE_ to the two separate experimental signatures for decay of the S_1_(ππ*) population, the agreement between their values confirms that they observe the same dynamical process of ^1^ππ* → ^1^nπ* relaxation within the adiabatic S_1_ state. The overall loss of S_1_ population is governed by direct internal conversion to the ground electronic state and ISC to T_2_, with negligible S_1_ → S_0_ radiative decay on the observed time scale of ∼12 ps. The kinetics of the GSB recovery (Figure 5c) indicate three distinct pathways for aqueous *p*-NP at pH/pD 5 or 3 to return to the ground state after near-UV photoexcitation. The branching ratios can be determined directly from the relative amplitudes of the three recovery components because all three processes are detected using changes in intensity of a single spectroscopic transition. Approximately 40% of the photoexcited *p*-NP undergoes the fastest recovery channel, with a time constant of ∼10 ps. Because of its rapidity, this channel is attributed to direct S_1_ to S_0_ internal conversion from either the ^1^ππ* or ^1^nπ* regions of the adiabatic S_1_ state. The recovery from ^1^ππ* and ^1^nπ*-character regions cannot be distinguished from analysis of the kinetics of the GSB recovery, because the GSB recovery time constant is largely controlled by the vibrational relaxation time constant for hot ground-state *p*-NP, which is typically ∼10 ps for an aromatic molecule in aqueous solution. From our TA measurements, the depopulation time constants of both the ^1^ππ* and ^1^nπ* regions of the S_1_ state can been separately determined: a decay time constant (τ_SE_ ≤ 200 fs) for dynamics away from the ^1^ππ* region is measured from the SE feature, and the decay time constant (τ_ISC_ = 12.2 ± 0.4 ps) for depopulation of the S_1_(nπ*) state is measured from its ESA feature. However, because the growth of the ^1^nπ* ESA may mask any evidence of prompt ^1^nπ* population decay back to S_0_ via a conical intersection on sub-ps time scales, we are unable to determine whether there is an ultrafast component of the S_1_/S_0_ internal conversion similar to that we recently reported for nitrobenzene photoexcited directly to its ^1^nπ* state in aqueous solution.^30^

**Table 1:** Kinetic Time Constants for Various TA Features for *p*-NP in pH 5 Aqueous Solution and 320 nm Photoexcitation

assignment	time constants	
S_1_(ππ*) (SE)	τ_SE_ ≤ 200 fs	-
S_1_(nπ*) (ESA)	τ_evo_ = 201 ± 22 fs	τ_ISC_ = 12.2 ± 0.4 ps
triplet state (ESA)	τ_ISC_ = 12.2 ± 0.4 ps	τ = 7.7 ± 0.4 ns^a^
ground state *p*-nitrophenolate band (400 nm), pH 5	τ_PT_ = 5.2 ± 0.1 ns (growth)	τ_Diff_ = 273.1 ± 8.8 ns
ground state *p*-nitrophenolate band (400 nm), pH 3	τ_PT_ = 4.9 ± 0.4 ns (growth)	τ_Diff_ = 28.5 ± 1.9 ns

a If, as argued here, ground state *p*-nitrophenolate is formed from the triplet state through ESPT and ISC, this time constant is expected to be similar to τ_PT_. The decay time constant of the triplet (1568 cm^–1^) band obtained in our TRIR measurement is 4.9 ± 0.1 ns, which matches well with τ_PT_. In TA spectra, the low intensity triplet band overlaps with the high intensity ground state *p*-nitrophenolate band, which might introduce error during decomposition of TA spectra that is reflected in the kinetics.

The remaining 60% of relaxation processes occur through the triplet manifold of excited states. ISC was observed from the S_1_(nπ*) state to the T_2_ (ππ*) state with τ_ISC_ = 12.2 ± 0.4 ps determined from loss of the S_1_(nπ*) ESA feature and growth of the T_1_ ESA band, as is shown in Figure 4b, followed by rapid relaxation to the ground vibrational level of the T_1_ state via IC and vibrational energy transfer to the solvent. A previous study (using a 267 nm excitation wavelength) suggested that this ISC occurs after excited-state proton transfer (ESPT).^25^ However, several reasons lead us to argue that ESPT occurs after ISC. Some of these reasons are based on the spectral assignments and mechanistic arguments presented above, which derive from the observed ∼12 ps ISC dynamics we identify from the S_1_ state of *p*-NP. If ESPT were to occur before ISC, producing the *p*-nitrophenolate in its S_1_ excited state, the subsequent transient behavior would match that revealed by TA studies of photoexcited *p*-nitrophenolate, which relaxes back to its ground (S_0_) state within a few picoseconds and without any evidence of ISC.^24^ Similar behavior was observed in our own TA and TRIR measurements for *p*-nitrophenolate (prepared from *p*-NP in pH/pD 13 aqueous solution) photoexcited at 400 nm, as detailed in Section S4 (Table S5 and Figures S14, S15, and S16) of the Supporting Information, which differs significantly from *p*-nitrophenol photochemistry. Prior studies of photoacid dynamics have established that the time constant for ESPT to the aqueous solvent depends on the strength of the photoacid in its excited state, ranging from some tens of nanoseconds for weak photoacids with p*K*_a_* ≈ 3 to subpicosecond for the strongest photoacid measured so far (with p*K*_a_ ≈ −8.5, for Quinone-cyanine 9 dye).^53^ Considering the structural similarity to phenol (which has p*K*_a_* = 5.7^54^), *p*-NP is expected to have p*K*_a_* ≥ 0, making it highly unlikely that ESPT could occur to the solvent within 12 ps.

A similar argument based on likely p*K*_a_* values supports our assignment of an ∼5 ns time constant (τ_PT_) to the ESPT process from the *p*-NP T_1_ state. As shown in Figure S17, the low value of the quantum-chemically calculated spin orbit coupling (0.06 cm^–1^) between the T_1_ (ππ*) and S_0_ states at the T_1_ minimum energy geometry disfavors ground-state *p*-NP recovery through T_1_/S_0_ ISC. Hence, the T_1_ state lifetime for aqueous *p*-NP is sufficient for ESPT to the solvent to occur. We note that this ESPT must form the conjugate base in a triplet state to conserve electron spin. As there are no features in our TA or TRIR spectra corresponding to intermediate species between the *p*-NP T_1_ state and ground-state (S_0_) *p*-nitrophenolate anion, the rate of any intermediate steps connecting the T_1_*p*-nitrophenolate anion and its ground S_0_ state must be faster than the 5 ns ESPT, making it impossible to determine precisely the pathway through which the S_0_ state of the *p*-nitrophenolate anion is reached. However, there are only two possibilities to consider: from the T_1_ anion state, ISC could populate the S_0_ state directly via a T_1_/S_0_ triplet-singlet crossing, or ISC could occur from the T_1_ to the S_1_ state of the anion, followed by rapid internal conversion to S_0_, as discussed in Section S4 of the Supporting Information. This second pathway is considered unfavorable because the ISC path connecting the T_1_ and S_1_ states is likely to be uphill in energy and therefore requires thermal activation, making it slow unless the S_1_ – T_1_ energy gap is small. The pH independence of τ_PT_ further supports our attribution of the ∼5 ns time constant to the ESPT step, with the subsequent ISC step being faster. If the ISC step were slower than ESPT, the T_1_ anion could reach an equilibrium with the *p*-NP T_1_ state, in which case the ESPT rate would become pH-dependent.

Under aqueous acidic pH (pH 5 and 3) conditions, the photogenerated *p*-nitrophenolate anions in their S_0_ state will combine with H^+^ ions available in the solution, completing the recovery of the ground state *p*-NP. This recombination can occur through two pathways: the first is rapid cage recombination, if an H^+^ ion is located within the first solvation shell of the *p*-nitrophenolate, and the second is slower diffusive nongeminate or geminate (after initial separation of the anion and proton) recombination. Under cage-recombination conditions, the rate-determining step for *p*-NP (S_0_) recovery remains the 5 ns step, during which *p*-nitrophenolate is formed by deprotonation of the *p*-NP T_1_ state, followed by deexcitation to the ground state of the anion. The second GSB recovery channel for *p*-NP, observed by TRIR and occurring on a comparable time scale, could therefore be assigned to this cage reprotonation. Alternatively, the assignment of this 7 ns GSB recovery could be a component of direct ISC from the T_1_ to S_0_ state of *p*-NP, similar to the T_1_/S_0_ ISC relaxation pathway identified for aqueous nitrobenzene.^30^ To explore this possibility further, we conducted TRIR experiments on aqueous (in D_2_O) *p*-NP solutions with addition of 1 M CsCl at pH 5. Our expectation was that if the 7 ns GSB recovery time constant were associated with any ISC process, then the rate would be enhanced in the presence of Cs^+^ ions, which are well-known to facilitate ISC.^55−57^ However, the nearly unchanged 7 ns GSB recovery time constants, as well as the *p*-nitrophenolate formation time constant (refer to Tables S4 and S6) in the presence of 1 M Cs^+^ leads us to favor the slow deprotonation and cage recombination process as the cause of the 7 ns *p*-NP (S_0_) recovery channel. The second, slower component of recombination between *p*-nitrophenolate anions and H^+^ ions is controlled by diffusion, representing the slowest recovery channel for aqueous *p*-NP after UV photoexcitation. From the decay time constants of the 400 nm bands observed by TA (see Table 1) and applying a pseudo first-order kinetic analysis, calculated rate constants for the reprotonation are 3.5 × 10^10^ and 5.2 × 10^10^ M^–1^ s^–1^ in pH 3 and 5 aqueous solutions, respectively. There is no evidence for photogenerated *p*-nitrophenolate in either the TA or TRIR spectra for aqueous solutions of *p*-NP at pH 1, which can be attributed to the faster diffusive recombination rate of the anion with H^+^. If we consider the diffusion-limited rate constant at pH 1 to be similar to those at pH 3 and pH 5 (i.e., ≥10^10^ M^–1^ s^–1^), the high H^+^ ion concentration at pH 1 would result in diffusive recombination between H^+^ ions and *p*-nitrophenolate with a time constant of ≤1 ns, which is faster than the ∼5 ns *p*-nitrophenolate formation time constant. However, the lifetime for decay of the T_1_ state at pH 1 remains similar to those observed at pH 3 and pH 5.

## Conclusions

Nitroaromatic compounds such as *p*-nitrophenol, nitrocatechols and nitromethoxyphenols are important organic constituents of brown carbon aerosols dispersed in the Earth’s troposphere. In large part because of these nitroaromatic chromophores, brown carbon aerosol particles absorb solar radiation at near-UV and visible wavelengths. Hence, they contribute to the radiative forcing of the atmosphere and to climate change, necessitating a better understanding of the optical properties and the photochemical aging of these particles when exposed to sunlight and to oxidizing species. The first steps of this photochemical processing are characterized here by investigating the consequences of near-UV photoexcitation of *p*-nitrophenol dissolved in aqueous solution. Using ultrafast TA and TRIR spectroscopy methods spanning time scales from 100 fs–8 μs, we successfully identify electronically excited state species and intermediates involved in a sequence of steps that ultimately recovers the *p*-nitrophenol parent molecule with high (∼100%) efficiency.

The *p*-nitrophenol molecule contains −OH and −NO_2_ substituents on an aromatic ring, and the UV photochemistry of both aqueous phenol and nitrobenzene were considered as models for interpretation of our measurements. On ultrafast (∼200 fs) time scales, the photoexcited *p*-NP state evolves in electronic character from ^1^ππ* to ^1^nπ* and then undergoes rapid ISC (∼12 ps) to populate its triplet (initially to the T_2_ state then to the T_1_ state via IC) excited state. In competition with ISC, internal conversion (IC) dynamics cause approximately 40% of the S_1_ (nπ*) state population to relax back to S_0_ and recover the parent *p*-NP (S_0_) after vibrational cooling through energy transfer to the solvent. These dynamics mirror those observed in aqueous nitrobenzene,^30^ as opposed to the autoionization of aqueous phenol (albeit after 267 nm UV excitation) to generate a solvated electron and the PhOH^+•^ radical cation, which in turn deprotonates to form a phenoxyl radical. Thereafter, the photochemical dynamics of the T_1_ state of *p*-nitrophenol deviate from those of nitrobenzene, as excited state proton transfer to the surrounding water produces triplet-state *p*-nitrophenolate which relaxes to its ground electronic state with overall ∼5 ns growth kinetics. The *p*-nitrophenolate anion is not observed in pH 1 solution because of rapid protonation, but at higher pH it is characterized by distinct absorption bands in the visible and mid-IR spectral regions that decay by cage-recombination and diffusion-limited protonation to recover *p*-nitrophenol.

Our extensive investigations of the pH-dependent photochemistry of this model nitroaromatic chromophore, using two complementary transient absorption spectroscopy methods over 6 orders of magnitude of time from 100 fs upward, provide clear evidence for the proposed mechanism and establish the time scales for multiple sequential photochemical and reactive steps. The deduced relaxation pathways should serve as guidance for efforts to unravel the photochemistry of other brown carbon chromophores such as nitrocatechols and nitromethoxyphenols containing both −NO_2_ and −OH functional groups. They also provide a mechanistic understanding of the very low quantum yields for UV photodegradation of aqueous nitrophenols,^15^ and the slow, inefficient photochemical processing of nitroaromatic compounds released from natural and anthropogenic sources into the environment. Our experimental studies used aqueous solutions that were not purged of dissolved oxygen, so the presence of oxygen does not suppress the recovery of the UV-photoexcited *p*-NP. However, in aqueous atmospheric aerosols containing other dissolved organic molecules, we cannot exclude the possibility of reactions of *p*-NP (T_1_) during its ∼5 ns lifetime leading to excited-state reactive degradation pathways.

## Experimental Section

Time-resolved spectroscopy measurements of aqueous *p*-NP photochemistry used both transient absorption (TA) with a white-light continuum (WLC) probe, and with a broadband mid-IR probe (time-resolved infrared spectroscopy, TRIR). Experiments were conducted using three separate ultrafast laser spectrometers located at the University of Bristol (UoB), University College London (UCL) and the Central Laser Facility at the STFC Rutherford-Appleton Laboratory (RAL). Brief descriptions are provided here of the three instruments used. The combination of these three setups provides wider spectral and temporal ranges for our TA measurements than are available from any one of the setups used alone. The additional spectral coverage is needed to observe all the reported ESA, SE, and *p*-nitrophenolate bands, while our combined experimental measurements also span time delays from 100 fs–8 μs. Furthermore, consistency of the results from the different TA Instruments serves to confirm the reliability of our measurements.

Wavelengths of 320 and 400 nm were employed to excite *p*-NP and *p*-nitrophenolate, respectively, in all three experimental setups. For TA measurements, a WLC was used to monitor changes in the population of ground and excited-state electronic energy levels after photoexcitation. For the UoB and UCL experiments, the WLC covered a wavelength range from 350–700 nm. At RAL, two different WLC probe pulses were used, covering the wavelength ranges from 360–470 and 500–875 nm. All the reported TRIR experiments were performed using the LIFEtime facility at RAL, with the same excitation wavelengths as for TA measurements. A pair of mid-IR probe pulses of ∼200 cm^–1^ bandwidth was used to cover the wavenumber range from 1400–1700 cm^–1^. The pump pulse energy was always set below 500 nJ to avoid undesired photoinduced processes. Further information is provided in Section S5 of the Supporting Information, and our previous publications report detailed descriptions of the TA set-ups at UoB^49,58^ and UCL,^30,59^ and the TRIR setup at RAL.^60,61^ The analysis of TA and TRIR data was performed using the KOALA software package^62^ for spectral decomposition, as described in Section S1 (contains Figure S4) of the Supporting Information. Briefly, this spectral decomposition resolved the different contributions to the TA spectra from broad and overlapping bands associated with excited-state absorptions, stimulated emission, and *p*-nitrophenolate ions. For the analysis of TRIR spectra, transient absorption and ground-state bleach features were fitted to Gaussian functions. In both cases, the wavelength-integrated intensities of the decomposed and fitted spectral components were plotted against time delay to extract excited-state lifetimes and the kinetics of the subsequent photochemical processes.

Aqueous sample solutions were prepared by dissolving commercial samples of *p*-NP (Sigma-Aldrich analytical standard) in deionized water or aqueous solutions with other pH values to typical concentrations of 2 mM. NMR spectroscopy of 2 and 10 mM samples confirmed that there was negligible aggregation of the *p*-NP solute molecules at these concentrations. Addition of aqueous hydrochloric acid or aqueous sodium hydroxide was used to control the pH of the solutions in the range pH 1–13. Given the weakly acidic nature of *p*-NP (p*K*_a_ = 7.2), its addition could potentially alter the pH, especially if the initial pH is close to the p*K*_a_ of *p*-NP. For example, when *p*-NP is added to deionized water to a concentration of 2 mM, partial dissociation results in an H^+^ concentration of 10^–5^ M (hence pH 5). We did not adjust such solutions to pH 7 by adding base to avoid the introduction of ions which could affect the results. TRIR experiments used solutions prepared in D_2_O (Sigma-Aldrich 99.9 atom % D) and acidified with aqueous DCl (Sigma-Aldrich ≥99 atom % D). Typically, 10 mL samples were continuously circulated through a Harrick cell fitted with CaF_2_ windows separated by 250 μm for TA and 100 μm for TRIR measurements. The combination of sample concentration and Harrick cell window separation was chosen to ensure the optical density at the UV excitation wavelength (usually 320 nm) was OD < 0.5.

## Calculations

The quantum chemistry method chosen to optimize the ground state structures and energies of *p*-nitrophenol and *p*-nitrophenolate was DFT/ωB97-X-D3,^63^ using the def2-SV(P) basis set for neutral species and the def2-TZVP basis set for charged systems. The ωB97-X-D3 functional was chosen as it is known to perform reliably for excited-state calculations of systems including weak solute–solvent interactions.^64,65^ To simulate the influence of solvent–solute interactions, an explicit water molecule was placed near each of the three oxygen atoms in *p*-nitrophenol, because these are the sites that will most readily accept hydrogen bonds from water molecules, and the entire system was reoptimized with the same functional and basis set in a PCM cavity. The optimized geometries are presented in Figure S18 of the Supporting Information. To describe the excited states, TDDFT within the Tamm–Dancoff approximation (TDA) with the same functional and basis set was employed. The vertical energies were modified using the (ptSS+LR)-PCM method, capable of accounting for the dynamical response of the fast component of the solvent polarization (ptSS) and excitonic coupling and dispersion effects (LR).^66^ The calculations were conducted with QChem 5.4. We employed the multilayer Multiconfigurational Time-Dependent Hartree (ML-MCTDH) method,^67^ implemented in Quantics,^68^ to simulate the nuclear quantum dynamics in full dimensionality (66 degrees of freedom for the microsolvated cluster in its neutral form). The potential energy surfaces were parametrized as a linear vibronic coupling model, taking gradients and nonadiabatic couplings at the Franck–Condon point and projecting them onto the normal mode coordinates of each system. Because of the computational cost, the nuclear quantum dynamics were propagated only up to 200 fs.

Quantum chemical calculations using linear-response time-dependent density functional theory (LR-TDDFT) were employed for the qualitative interpretation of the photoinduced processes occurring on time scales >200 fs. Geometry optimizations of ^1^nπ* and T_1_ minima were conducted in Gaussian 16 (RevA.03)^69^ using the ωB97-X-D^70^ functional and def2-SV(P)^71^ basis set with implicit solvation by a water PCM. Frequency calculations were performed to confirm the identity of minima. A geodesic interpolation was used to generate 10 intermediate geometries between the S_0_ minimum and ^1^nπ* and T_1_ minima.^72^ At each of these intermediate geometries, (LR-TD)DFT/TDA/ωB97-X-D/ZORA-def2-SV(P) with a water CPCM (conductor-like polarizable continuum model) was used to calculate energies of excited states and the spin–orbit coupling (SOC) between singlet and triplet states. These calculations were performed using ORCA 5.0.3^73,74^ with the zeroth order regular approximation (ZORA) to the Dirac equation for scalar relativistic effects.^75^ The SOC magnitudes reported in this work were calculated for each molecular structure using eq 1 to account for all the triplet sublevels.
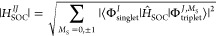
1

## Data Availability

Data are available at the University of Bristol data repository, data.bris, at 10.5523/bris.3pktsqxqmh5772c7uhvbiwccpb.

## Supporting Information Available

Additional details from the quantum chemical calculations include electronic transitions, HOMO–LUMO diagrams, optimized geometries, quantum dynamic results, and results obtained from geodesic interpolation in internal coordinates between different geometries; additional TA and TRIR data, decomposition of TA and TRIR spectra, and kinetics obtained after decomposition are also provided; a detailed description of the TA setups at UoB and UCL, as well as the TA and TRIR experiments at RAL, is included; additionally, thermodynamic considerations of the ionization of *p*-NP are discussed (PDF)
