# Supplementary material for: Efficient Ground-State Recovery of UV-Photoexcited p-Nitrophenol in Aqueous Solution by Direct and Multistep Pathways
Source: J Am Chem Soc. 2024 Oct 25;146(44):30443–54. doi: 10.1021/jacs.4c10965 (PMC11544619; doi:10.1021/jacs.4c10965)
Supplement: Supplementary file 1 — ja4c10965_si_001.pdf [file ja4c10965_si_001.pdf]

Supporting Information for: Efficient ground-state recovery of UV-photoexcited p-nitrophenol in aqueous solution by direct and multi-step pathways

Deborin Ghosh,[a,*] K. Eryn Spinlove,[b] Hallam J.M. Greene,[a] Nicholas Lau,[b] Sandra Gómez,[b,c] Min-Hsien Kao,[a] William Whitaker,[a] Ian P. Clark,[d] Partha Malakar,[d] Graham A. Worth,[b] Thomas A. A. Oliver,[a] Helen H. Fielding[b] and Andrew J. Orr-Ewing[a,*]

[a] *School of Chemistry, University of Bristol, Cantock's Close, Bristol BS8 1TS, UK*

[b] *Department of Chemistry, University College London, 20 Gordon Street, London WC1H 0AJ, UK*

[c] *Departamento de Química Física, Universidad de Salamanca, Salamanca, 37008, Spain*

[d] *Central Laser Facility, Research Complex at Harwell, Science and Technology Facilities Council, Rutherford Appleton Laboratory, Harwell Oxford, Didcot, Oxfordshire, OX11 0QX, UK*

* Authors for correspondence: deborin.ghosh@bristol.ac.uk, a.orr-ewing@bristol.ac.uk

Contents **Page**

Supplementary Figures

Figure S1 S2
Figure S2 S3
Figure S3 S4
Figure S4 S5
Figure S5 S6
Figure S6 S6
Figure S7 S7
Figure S8 S8
Figure S9 S10
Figure S10 S11
Figure S11 S12
Figure S12 S12
Figure S13 S12
Figure S14 S13
Figure S15 S14
Figure S16 S15
Figure S17 S16
Figure S18 S20

Supplementary Tables

Table S1 S3
Table S2 S7
Table S3 S9

Table S4 S9
Table S5 S13
Table S6 S15

Supplementary Sections

Section S1: Decomposition of TA and TRIR Spectra S4
Section S2: Photochemical pathways of p-NP in Chloroform ($CHCl_3$) and Acetonitrile (MeCN) solutions S8
Section S3: Thermodynamic considerations of ionization of p-NP S9
Section S4: p-nitrophenol in an alkaline aqueous solution S13
Section S5: Experimental Methodology S17
References S20

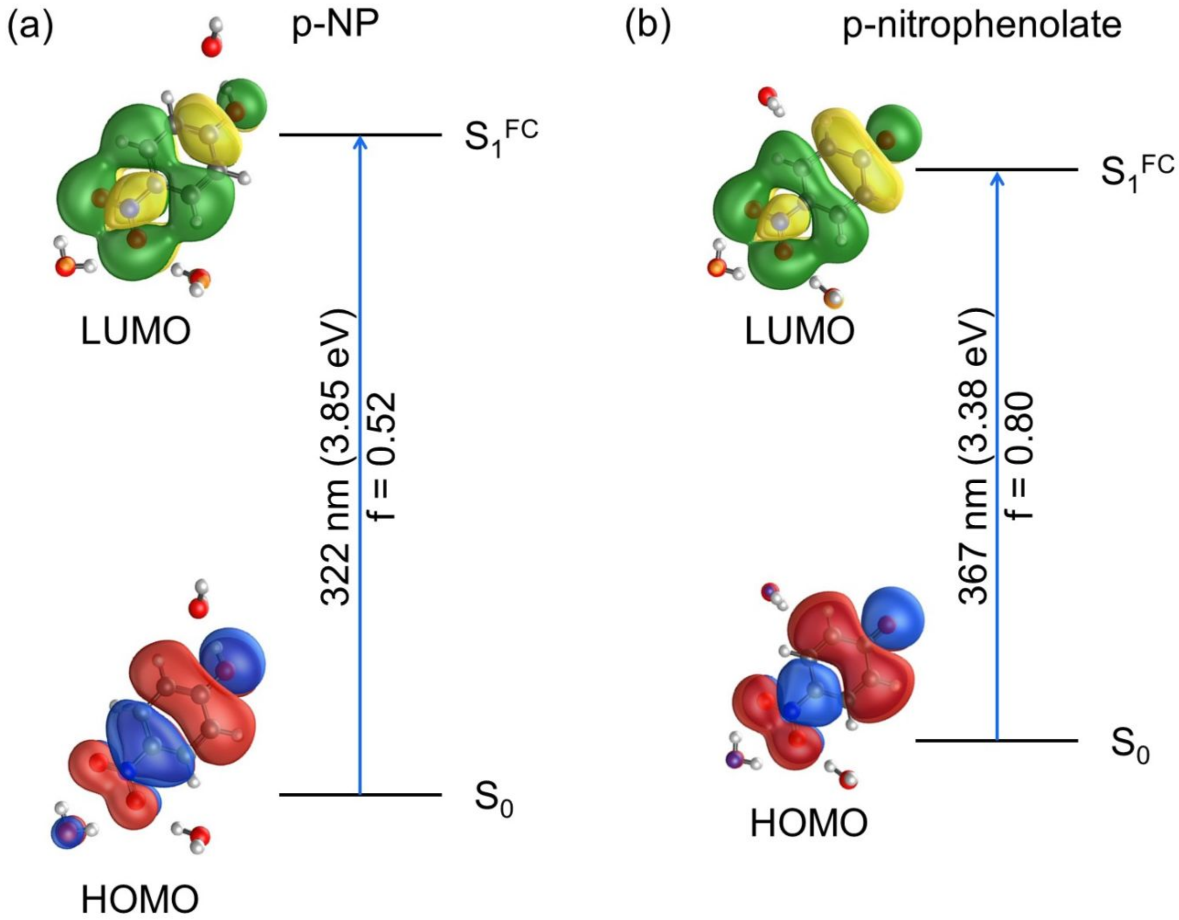

Figure S1: Energy level diagrams for (a) microsolvated p-NP and (b) microsolvated p-nitrophenolate in a water (PCM) cavity. Microsolvation is by 3 explicit water molecules. The TDA-TDDFT/ωB97-X-D3/def2-SV(P) method was used to calculate the vertical energies of the p-NP, whereas for p-nitrophenolate the basis set was enlarged to def2-TZVP. In the figure, f denotes the calculated oscillator strength.

Table S1: The first few calculated Franck–Condon excited energy levels of p-NP and p-nitrophenolate, micro-solvated by three water molecules in a water (PCM) cavity, along with their character and transition energies and oscillator strength. The TDA-TDDFT/ωB97-X-D3/def2-SV(P) method was used to calculate the vertical energies of the p-NP, whereas for p-nitrophenolate the basis set was enlarged to def2-TZVP.

**Molecule**	**Franck–Condon (FC) excited electronic state and their character**	**Transition energies (Oscillator strength)**
p-NP	S_1 ($\pi_{AQ}\pi_1$*)	3.85 eV / 322 nm (0.52)
	S_2 ($n_1\pi_1$*)	4.26 eV / 291 nm (0.00)
	S_3 ($\pi_Q\pi_1$*)/($\pi_{AQ}\pi_2$*)	4.67 eV / 265 nm (0.00)
	S_4 ($n_2\pi_1$*)	4.85 eV / 255 nm (0.00)
p-nitrophenolate	S_1 ($\pi_{AQ}\pi_1$*)	3.38 eV / 367 nm (0.80)
	S_2 ($n_{an}\pi_1$*)/($n_1\pi_1$*)	3.94 eV / 315 nm (0.00)
	S_3 ($n_1\pi_1$*)/($n_{an}\pi_1$*)	4.42 eV / 280 nm (0.00)
	S_4 ($\pi_Q\pi_1$*)/($\pi_{AQ}\pi_2$*)	4.73 eV / 262 nm (0.01)

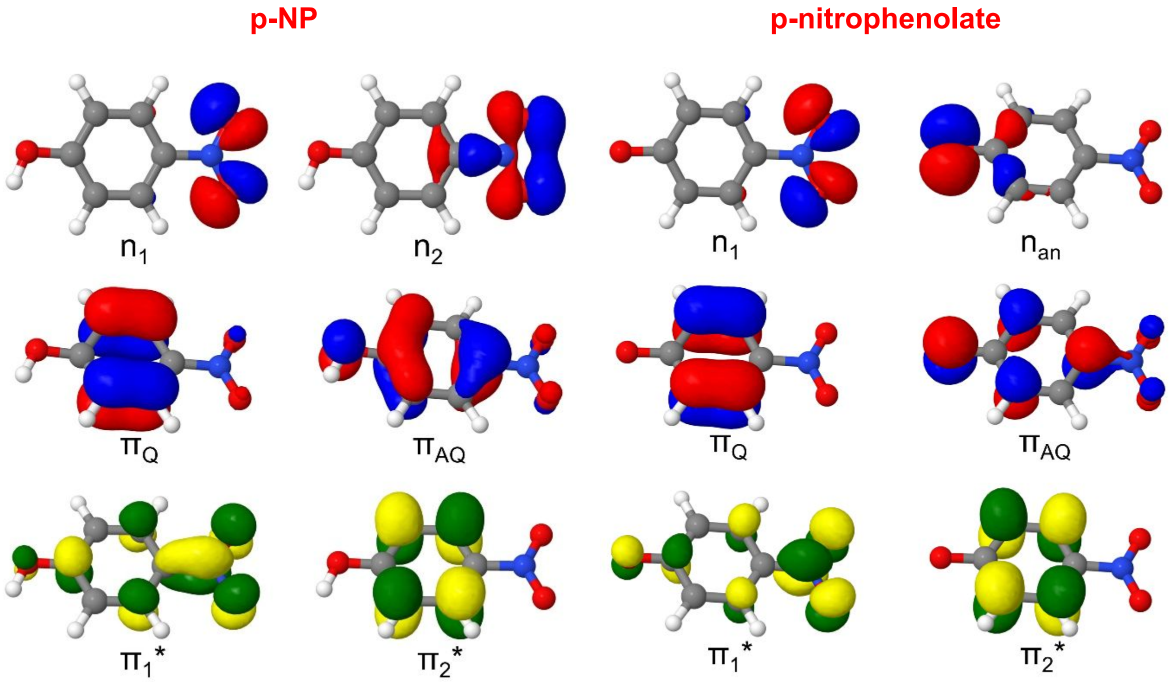

Figure S2: Molecular orbitals for p-nitrophenol and p-nitrophenolate calculated at the DFT ωB97-X-D3/def2-SV(P) level of theory.

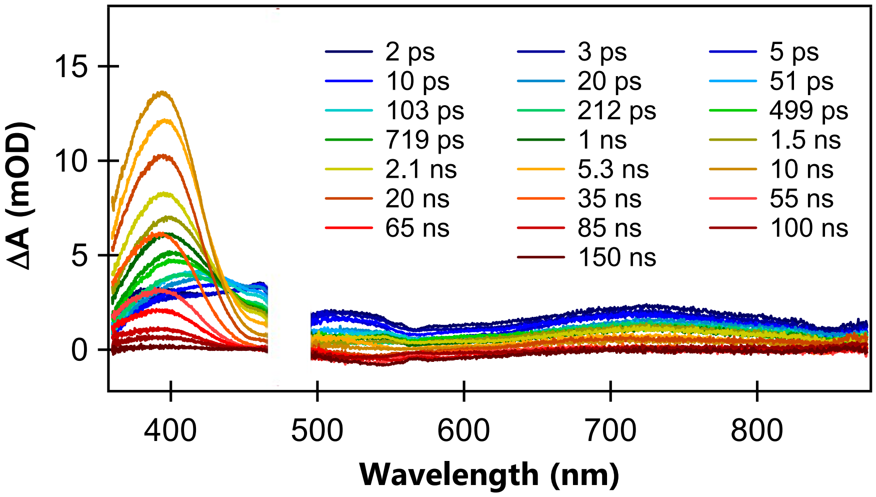

Figure S3: TA spectra of p-NP at different time delays in a pH 3 aqueous solution, obtained using the LIFEtime TA spectroscopy instrument at RAL. 320 nm pump light was used for photoexcitation. The inset key identifies the colours used to plot TA spectra obtained at different time delays. The white box covers an area between the WLC generated with 1030 nm and 515 nm pump pulses (see Section S5.3) for which the WLC intensity is too low for spectral measurements.

Section S1: Decomposition of TA and TRIR Spectra

The decomposition of all TA and TRIR data was conducted using the KOALA program.[8] Gaussian functions or TA spectra obtained for specific time-delays were employed as fitting functions to fit various TA or TRIR features for aqueous p-NP. Figure S4(a)-(f) illustrates the decomposition of TA and TRIR spectra at different time-delays.

In Figure S4(a), stimulated emission (SE) from the $S_1(^1\pi\pi^*)$ state and excited state absorption (ESA) from the $S_1(^1n\pi^*)$ state were observed at 250 fs. Figure S4(b) shows the decomposition of the TA spectrum at ~10 ps into ESA from the $S_1(^1n\pi^*)$ state and ESA from the triplet state. The TA spectrum at 2 ns is a combination of ESA from the triplet state and ground state absorption (GSA) by p-nitrophenolate anions (see Figure S4(c)). A floating Gaussian function was employed to fit the ESA from the triplet state up to 50 ps. This choice was made due to the continuous shift to shorter wavelength observed in the TA spectra with time delay. This spectral shift is attributed to internal conversion (IC) to the T_1 state from a higher-lying triplet state initially populated by ISC from S_1 (in accord with El Sayed's rules[11, 12]) and vibrational cooling in T_1. Subsequently, the TA spectrum at 50 ps was used as the fitting function for the triplet ESA component.

In Figure S4(d), four Gaussian functions were employed to model the TRIR data at 10 ps. Two of these functions correspond to the ground state bleaches (GSB) at 1597 and 1610 cm^{-1}, while the other two represent excited state vibrational absorption bands originating from the T_1 triplet state and a feature corresponding to hot ground state (S_0) absorption (HGSA). The HGSA exhibited a continuous shift towards higher energy with increasing time-delay, consistent with vibrational cooling in S_0. Figure S4(f) presents an alternative approach, wherein the Gaussian function used for fitting the ground state absorption (GSA) from p-nitrophenolate was replaced by the steady-state Fourier-transform infrared (FTIR) spectrum of p-nitrophenolate.

The integrated areas of the fitted functions describe the band intensities at each time delay, and changes in these intensities over time reveal the kinetics of each transient feature.

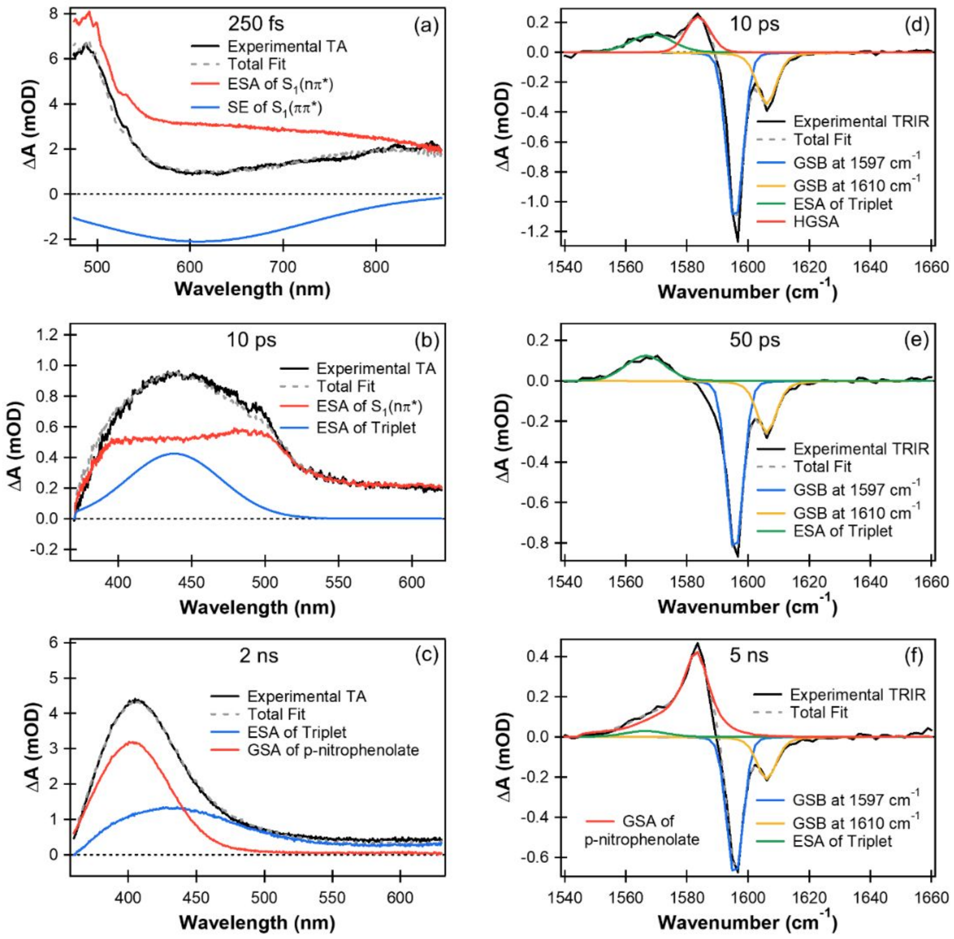

Figure S4: Spectral decomposition of features in the TA and TRIR spectra for aqueous solutions of p-NP photoexcited at 320 nm. The panels show representative (a)-(c) TA and (d)-(f) TRIR spectra of p-NP at pH 5 at selected time delays, along with the decomposition into various transient features discussed in the main paper. The inset keys identify the experimental spectra, the fit functions used and the resulting fits.

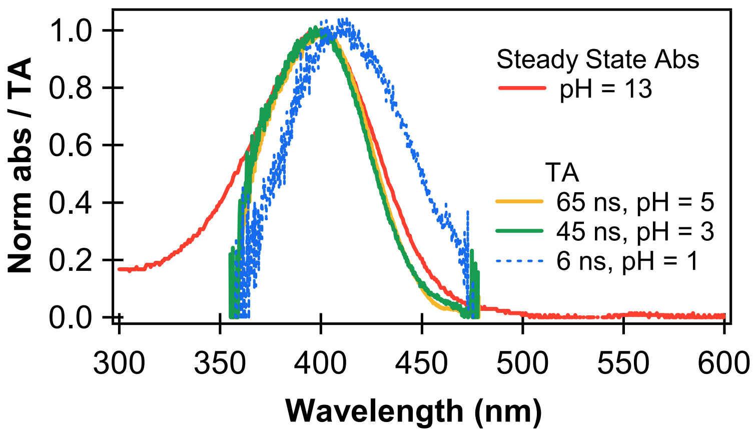

Figure S5: Normalized 400 nm TA bands for p-NP in pH 1, 3, and 5 aqueous solutions obtained at time delays of 6, 45 and 65 ns, respectively. A steady state UV-Vis absorption spectrum of aqueous p-nitrophenolate (red solid line) has been added for comparison. At pH 5 and pH 3, the TA band is assigned to the p-nitrophenolate anion. The shifted band observed at pH 1 is instead assigned to excited state absorption from the T_1 state of p-NP.

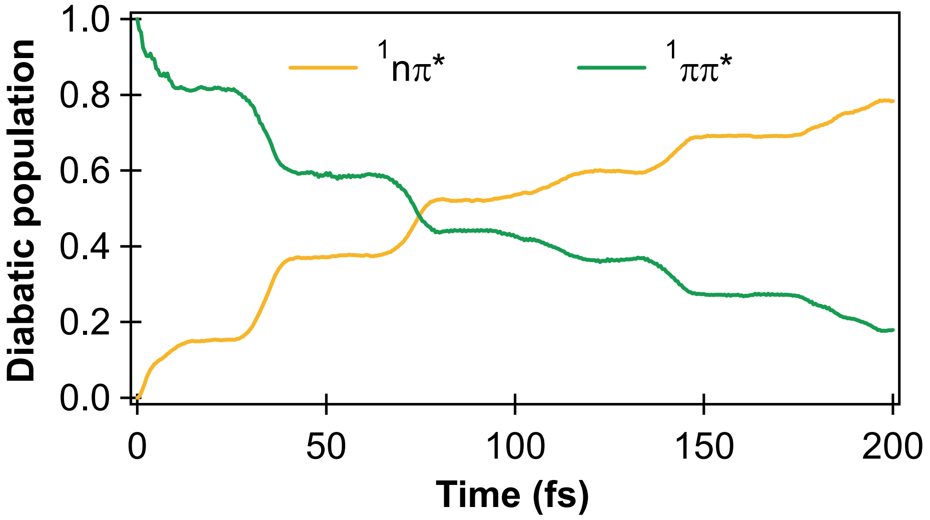

Figure S6: ML-MCTDH simulated time-dependence of the diabatic populations of the microsolvated photoexcited p-NP.

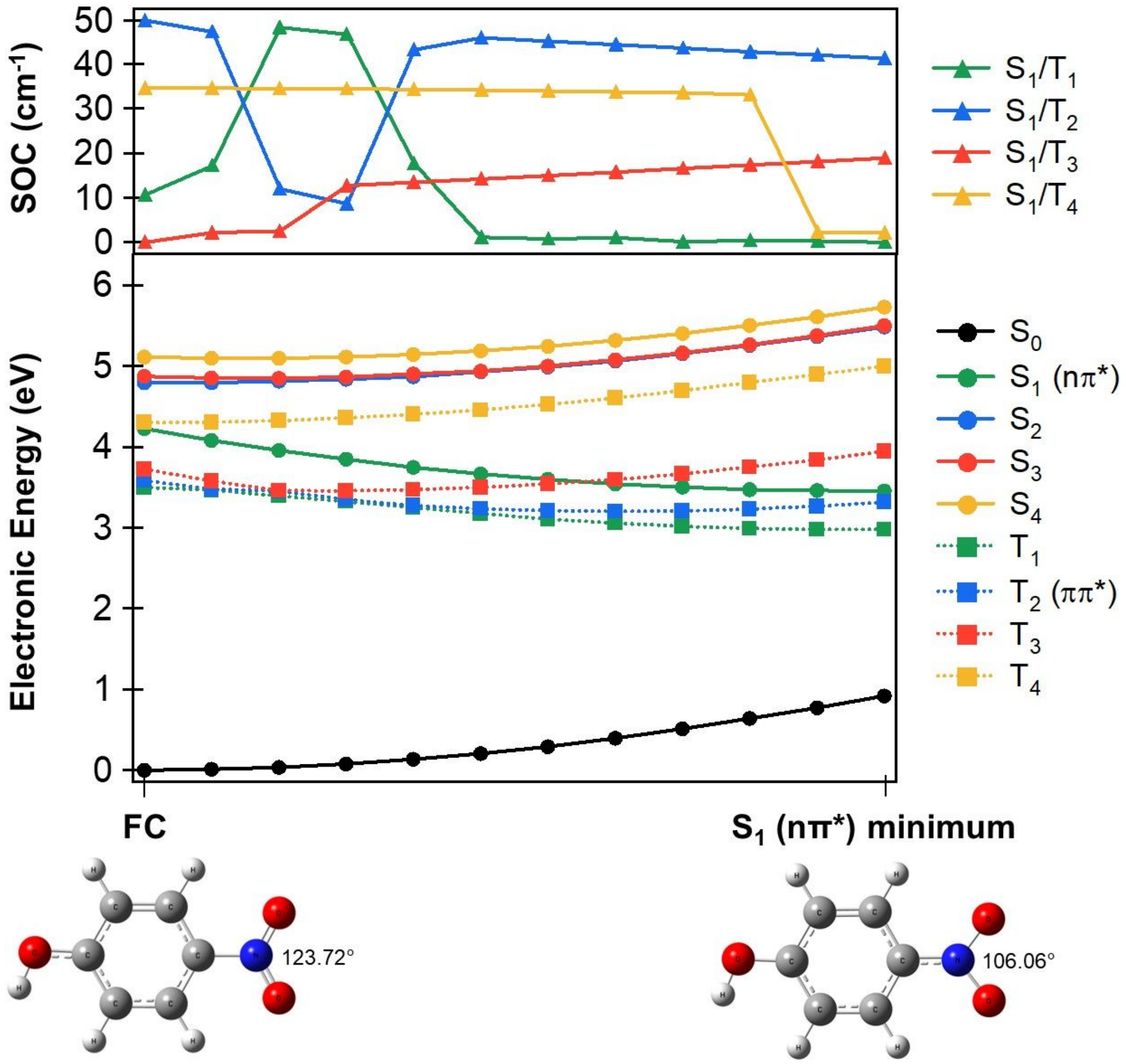

Figure S7: A geodesic interpolation using 10 intermediate steps between geometries of the ground state minimum (left hand side geometry) and S_1(nπ*) minimum (right hand side geometry). Energies of the ground and excited states relative to the S_0 minimum (lower panel) and the S_1-T_n spin orbit coupling (SOC) at each geometry (upper panel) are calculated using (LR-TD)DFT/TDA/ωB97-X-D3/ZORA-def2-SV(P) with a non-equilibrium CPCM.

Section S2: Photochemical pathways of p-NP in Chloroform ($CHCl_3$) and Acetonitrile (MeCN) solutions

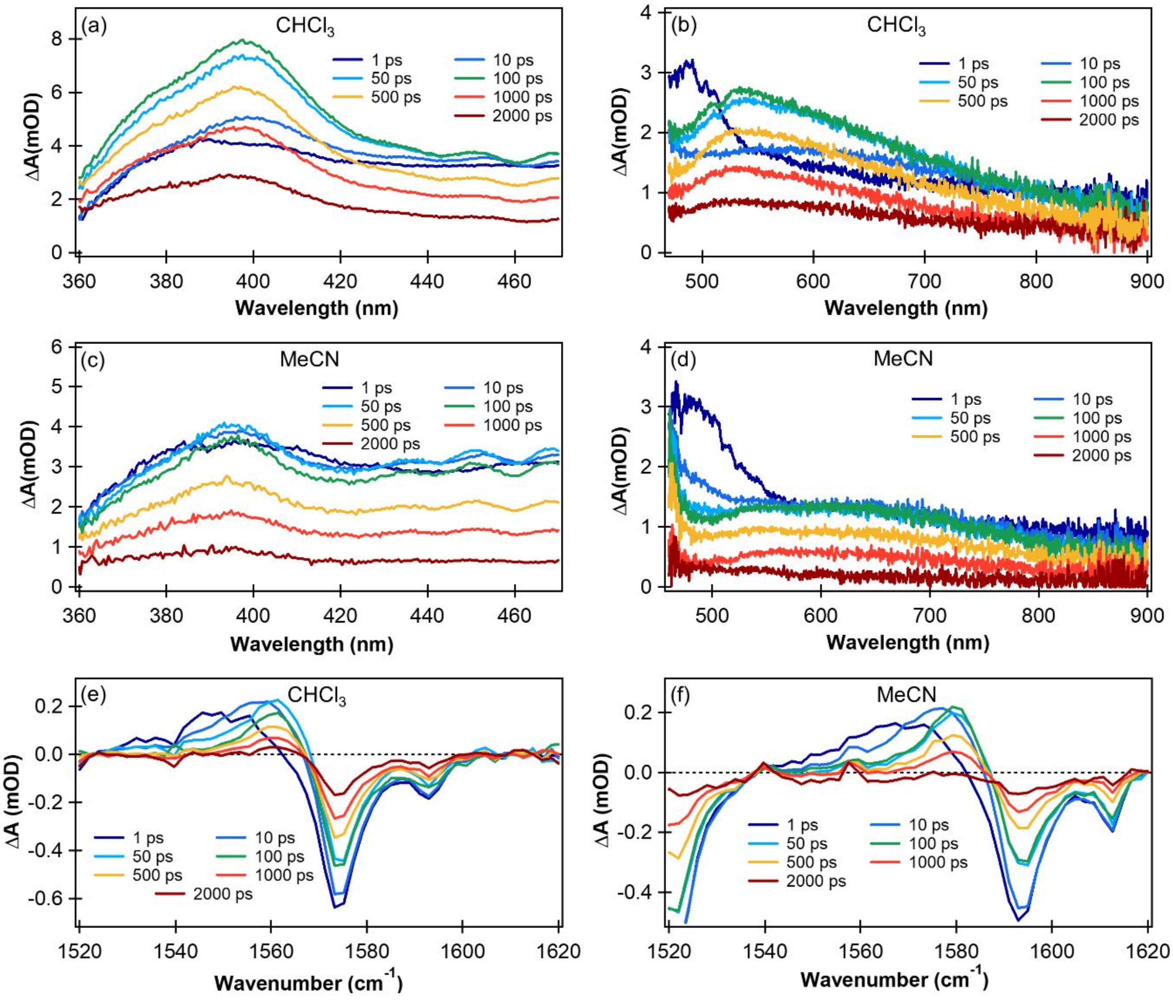

Figure S8: TA and TRIR spectra of p-NP in $CHCl_3$ and MeCN solutions photoexcited at 320 nm. (a) and (b) TA spectra of p-NP in $CHCl_3$ in the 360 – 470 nm and 480 – 900 nm probe windows, respectively. (c) and (d) TA spectra of p-NP in MeCN in the 360 – 470 nm and 480 – 900 nm probe windows, respectively. (e) and (f) TRIR spectra of p-NP in $CHCl_3$ and deuterated MeCN respectively.

Two distinct features are observed from the TA and TRIR spectra of 320-nm photoexcited p-NP in $CHCl_3$ and MeCN (deuterated MeCN for TRIR). A broad absorption band at 390 nm was observed for p-NP in $CHCl_3$ and MeCN at very early times in the 360 – 470 nm probe window, and then it evolved into a more structured band. Also, in the 480 – 900 nm probe window, a peak at 490 nm was observed at early times which then disappeared quickly, while a broad feature centred around 550 nm in $CHCl_3$ and around 600 nm in MeCN evolved.

Similar time-dependent behavior was observed in the TRIR spectra for these p-NP solutions. A broad ESA band around 1549 cm^{-1} in $CHCl_3$ and 1567 cm^{-1} in deuterated MeCN at early times evolved into a sharp ESA band around 1560 cm^{-1} and 1579 cm^{-1} in $CHCl_3$ and deuterated MeCN, respectively. The kinetics of the changes in peak shapes are similar to those for the $^1n\pi^*$ to triplet state ISC observed in aqueous media. Unlike in aqueous solutions, the triplet state decays back to the ground state without any evidence for ESPT in $CHCl_3$ and MeCN. The photochemical behaviour of p-NP in $CHCl_3$ and MeCN (i.e. in non-aqueous solutions) is therefore similar to the photochemical behaviour we recently reported for nitrobenzene.[1]

Section S3: Thermodynamic considerations of ionization of p-NP

Recommended values of oxidation potentials for phenol (PhOH) and p-NP referenced to a standard hydrogen electrode (SHE) have recently been reported,[2] and are listed in Table S2. To estimate threshold wavelengths corresponding to the thermodynamic limits for autoionization of photoexcited phenol and p-NP in aqueous solution, we have converted these oxidation potential values to aqueous (solvated) electron references which are also presented in Table S2. This conversion used a Gibbs energy value for an electron in the SHE relative to vacuum of 428.4 kJ mol^{-1} [3] and a value for the hydration Gibbs energy (from vacuum) of an electron of -148.5 kJ mol^{-1}.[4] From these corrected potentials, we calculated the Gibbs energy (ΔG) changes for ionization in aqueous solution, and using ΔG, we determined the threshold wavelengths for UV ionization for aqueous solutions of phenol and p-NP (see Table S2).

Table S2: Various thermodynamic parameters for the ionization reaction of PhOH and p-NP.

Reaction	Oxidation potential (V) vs SHE[2]	Corrected oxidation potential (V) for e_{aq}^-	ΔG (kJ mol^{-1})	Threshold wavelength (nm)
**$PhOH \rightarrow PhOH^+ + e^-$**	0.874 – 1.087	3.77 – 3.99	364.2 – 384.7	328 - 311
**$p\text{-NP} \rightarrow p\text{-NP}^+ + e^-$**	1.165 – 1.328	4.07 – 4.23	392.3 – 408.0	305 - 293

The threshold wavelength for UV ionization for PhOH is longer than the absorption maximum of aqueous PhOH, which is 270 nm. The formation of a solvated electron by autoionization has been observed after photoexcitation of PhOH with 267 nm and 200 nm pulses.[5] However, the reported formation time scales of solvated electrons were very different for short (200 nm) and long (267 nm) excitation wavelengths (femtosecond and nanosecond, respectively).[5]

On the other hand, the threshold wavelength of ionization for p-NP is at least 15 nm shorter than the absorption maximum (320 nm) in an aqueous medium. Hence, energetic

considerations dictate that a solvated electron band should not arise following photoexcitation of aqueous p-NP at wavelengths longer than the band maximum. Nevertheless, we observed similar TA spectra (all featuring a broad absorption band centered around 700–800 nm) and kinetics for p-NP photoexcited at 310, 320, and 360 nm wavelengths (See Figure S9 and Table S3). Drawing on the example of phenol autoionization timescales, the observed rate of growth of this band is implausibly rapid for p-NP autoionization at these excitation wavelengths. Therefore, assignment of the broad TA absorption band centered around 700-800 nm to a solvated electron band is discounted.

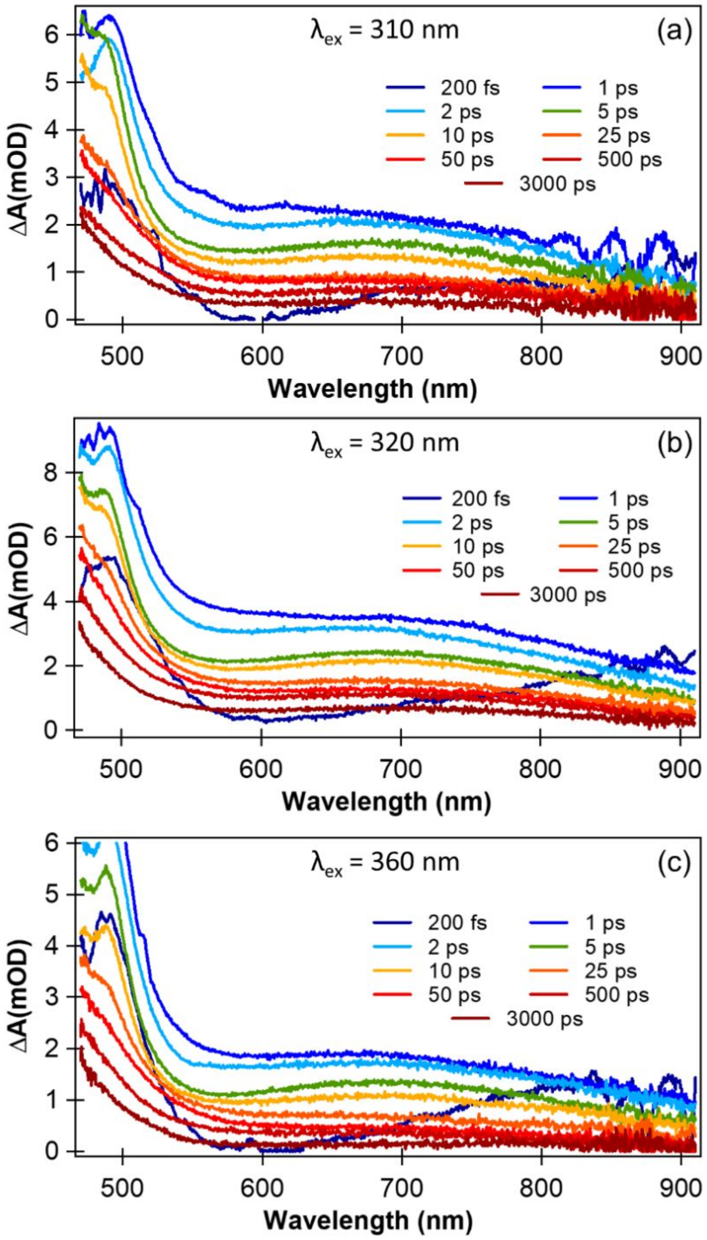

Figure S9: TA spectra for p-NP pH 5 aqueous solutions obtained using different excitation wavelengths (a) 310 nm, (b) 320 nm and (c) 360 nm.

Table S3: Kinetic time constants of the broad absorption band centered around 700–800 nm of p-NP in pH 5 at different excitation wavelengths.		
**Excitation wavelength (nm)**	τ_1	τ_2
**310**	8.20 ± 0.49 ps	4.11 ± 0.33 ns
**320**	8.37 ± 0.53 ps	4.45 ± 0.32 ns
**360**	11.56 ± 0.55 ps	3.06 ± 0.36 ns

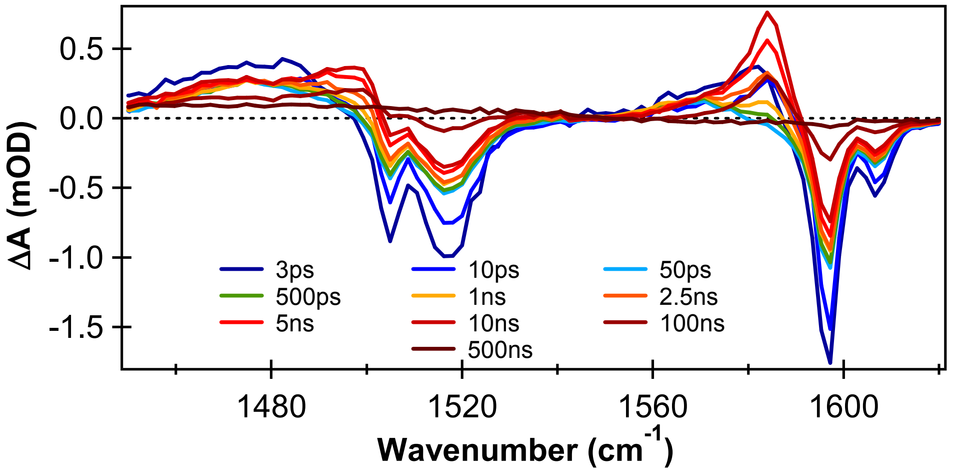

Figure S10: TRIR spectra of p-NP in D_2O at pD 3 for different time delays. 320 nm pump light was used for photoexcitation. The inset colour key identifies TRIR spectra obtained at different pump-probe time delays.

Table S4: Ground state bleach (GSB) recovery kinetic time constants extracted from TRIR measurements for p-NP in aqueous solutions of different pD. The relative amplitudes of the different growth or decay components are shown as percentages in parentheses.			
**pD**	**τ_1 (ps)**	**τ_2 (ns)**	**τ_3 (ns)**
**1**	10.1 ± 0.9 (37 %)	5.2 ± 0.2 (63 %)	-
**3**	9.8 ± 0.6 (40.2 %)	6.2 ± 0.6 (24.1 %)	109 ± 7 (35.7 %)
**5**	10.2 ± 0.6 (39.5 %)	7.2 ± 0.5 (26.5 %)	180 ± 10 (34 %)
**5, 1M CsCl**	9.1 ± 0.8 (45 %)	7.0 ± 1.0 (23 %)	567 ± 57 (33 %)
(5, 1M CsCl) represents 1M CsCl added to pD 5 solution.			

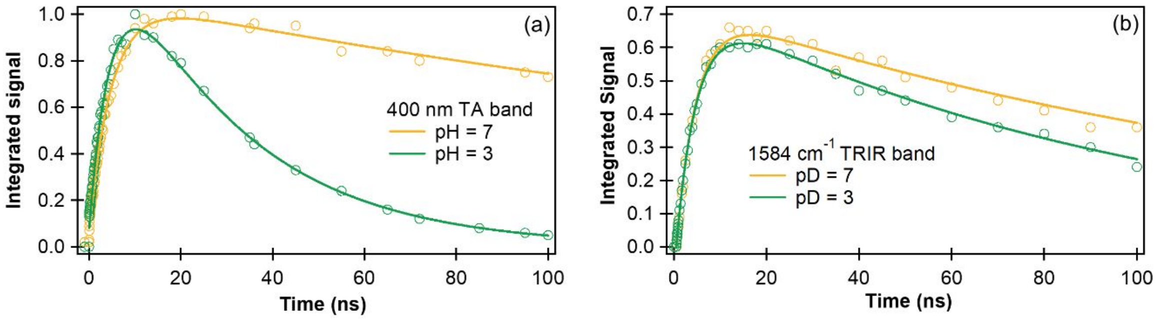

Figure S11: Kinetics of (a) the 400 nm TA band and (b) the 1584 cm^{-1} TRIR band of aqueous p-NP in pH/pD 3 and 5 solutions photoexcited at 320 nm, derived from spectral decomposition of TA and TRIR spectra. The TA and TRIR bands are both assigned to aqueous p-nitrophenolate.

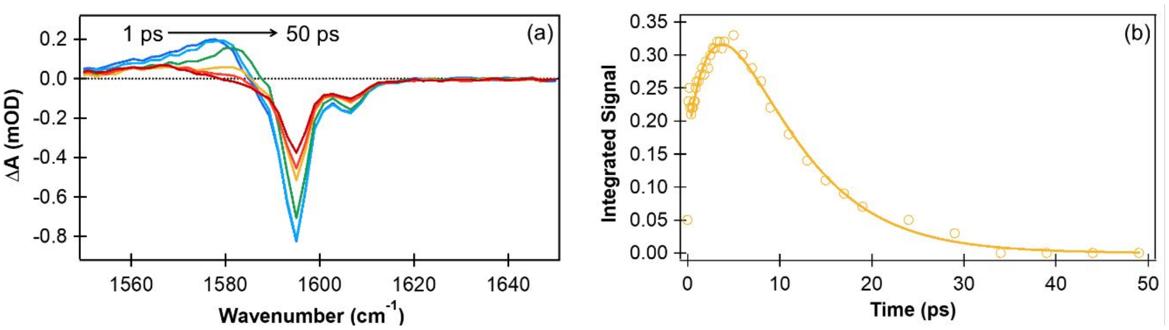

Figure S12: (a) TRIR spectra and (b) kinetics of the hot ground state absorption (HGSA) band of 320-nm photoexcited p-NP in pD 5 aqueous (D_2O) solution.

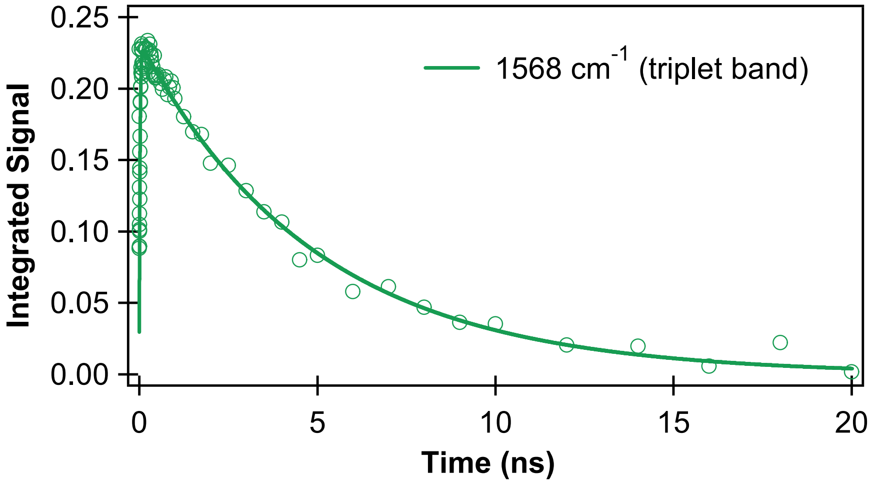

Figure S13: Kinetics of the 1568 cm^{-1} TRIR band (which is assigned as an ESA vibrational band of the triplet state) for p-NP in D_2O at pD 5.

Section S4: p-nitrophenol in an alkaline aqueous solution

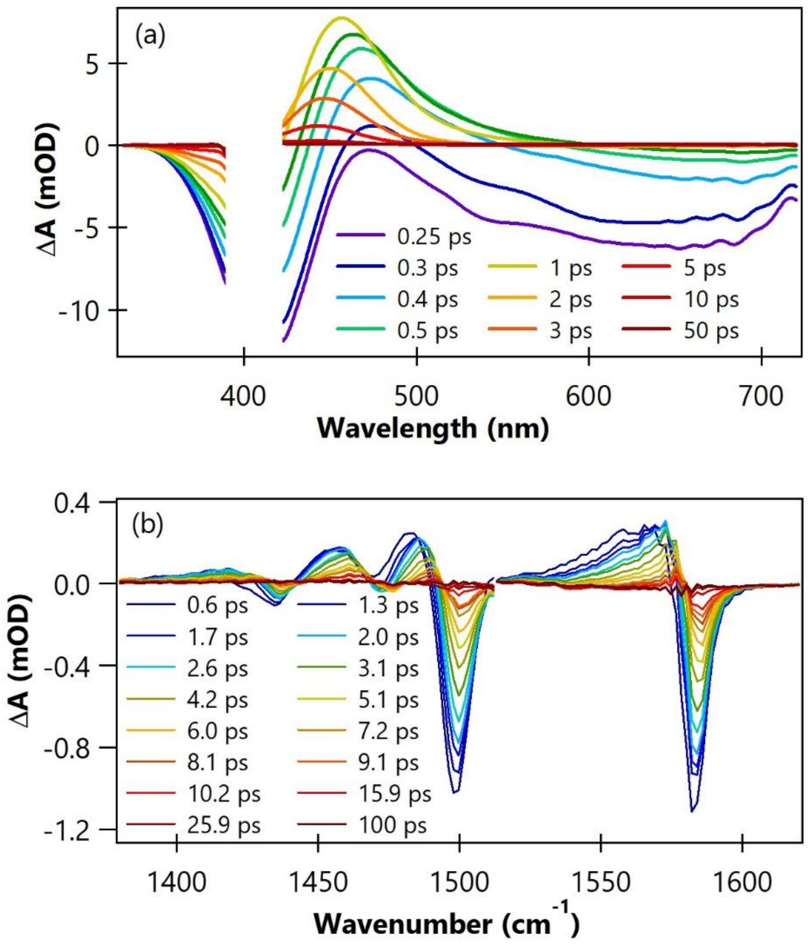

Figure S14: (a) TA and (b) TRIR spectra of aqueous p-nitrophenolate at different time delays in pH/pD 13. In all the measurements, 400-nm UV pump light was used for photoexcitation.

TA and TRIR spectra of p-nitrophenolate are presented in Figure S14. The photochemical behaviour of p-nitrophenolate in aqueous and non-aqueous media has already been described by Michenfelder *et al.*[6] Though different methods of spectral fitting have been used, we observe similar kinetics to their report. We have also taken TRIR absorption measurements, and the kinetics obtained from the TRIR spectra support the photochemical behaviour of p-nitrophenolate deduced from TA measurement. Unlike the previous report, a clear GSB signal around 400 nm is observed in our TA spectra, although it is partially masked by pump-beam scatter. As we used Gaussian basis functions to fit the transient spectra, the pump scattering at 400 nm (dashed rectangle) did not present a major difficulty for the analysis. Ground state bleach recovery could also be determined from 1584 cm^{-1} and 1495

cm^{-1} GSB bands in the TRIR spectra. However, we could not separately identify the ESA in our analysis. We also observed a broad negative feature at very early time points in TA spectra corresponding to SE, with a decay time constant of 0.16 ± 0.01 ps which is similar to the previous report. The torsional motion of the NO_2 group in the S_1 state which was reported to account for the broad nature of the SE bands leads to a conical intersection between the S_1 and S_0 states.[6] SE and the IC through the conical intersection are responsible for building population in the higher vibrational levels of the S_0 state. A positive growing feature at early times very close to the GSB signal and a continuous higher energy shift due to vibrational relaxation are the key characteristics of absorption from higher vibrational levels of the ground state (hot ground state absorption, HGSA). The kinetics of this HGSA can be extracted from both our TA and TRIR absorption spectra. An initial growth (time constant 0.25 ± 0.01 ps) with a similar time constant of SE decay, followed by a mono-exponential decay is observed. The decay time constant of the HGSA signal is similar to the GSB recovery time constant which indicates that there are no other deexcitation processes involved in an aqueous medium. All the kinetics obtained from the analysis of TA and TRIR spectra are presented in Figure S15 and the time constants are reported in Table S5. All the processes involved after photoexcitation of the p-NP anion are summarized in a schematic energy profile diagram in Figure S16.

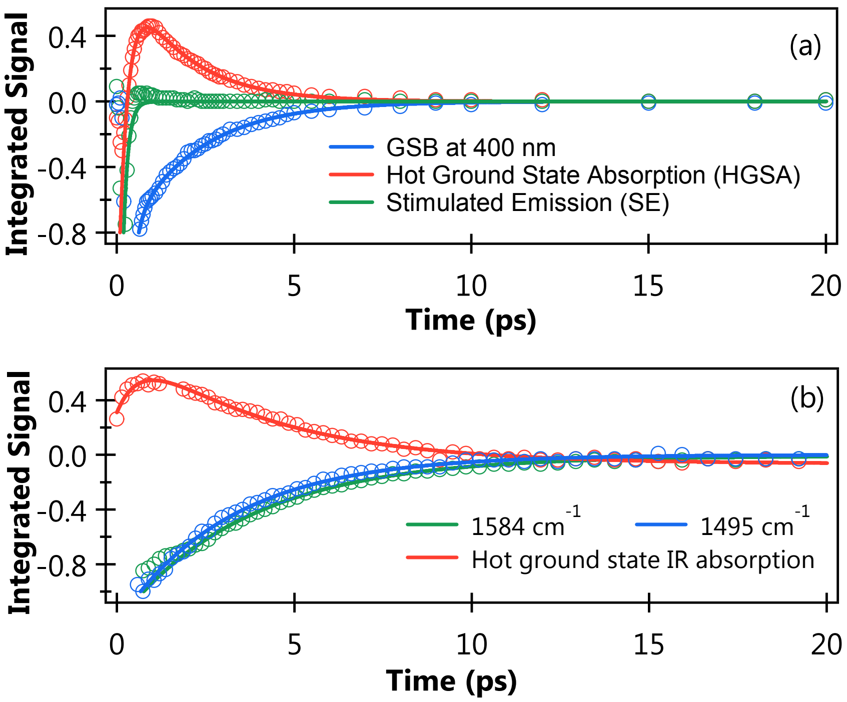

Figure S15. (a) Kinetics of GSB recovery, SE, and HGSA obtained from TA spectra of aqueous p-nitrophenolate photoexcited at 400 nm. (b) Kinetics of GSB recovery of 1584 and 1495 cm^{-1} bands and a HGSA absorption band obtained from TRIR spectra of p-nitrophenolate.

Table S5: Time constants for different photoinduced processes for aqueous solutions of p-nitrophenolate.		
**Process**	**Band**	**Time constant (τ)**
GSB recovery	400 nm	1.99 ± 0.04 ps
	1584 cm^{-1}	3.63 ± 0.05 ps
	1495 cm^{-1}	3.07 ± 0.05 ps
SE	Broad band around 650 nm	0.16 ± 0.01 ps
HGSA	400-500 nm	0.25 ± 0.01 ps (growth), 1.68 ± 0.03 ps
	1550-1600 cm^{-1}	0.69 ± 0.08 ps (growth), 3.94 ± 0.20 ps

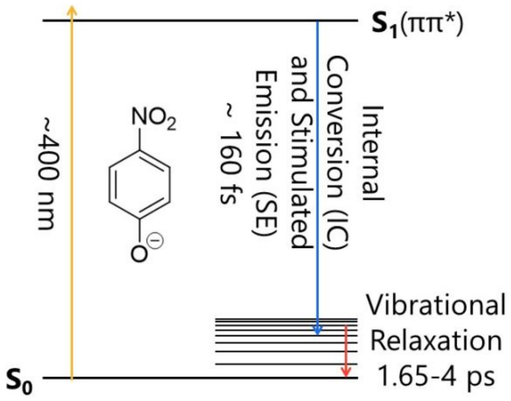

Figure S16: Schematic energy profile diagram of p-nitrophenolate indicating all involved processes after photoexcitation.

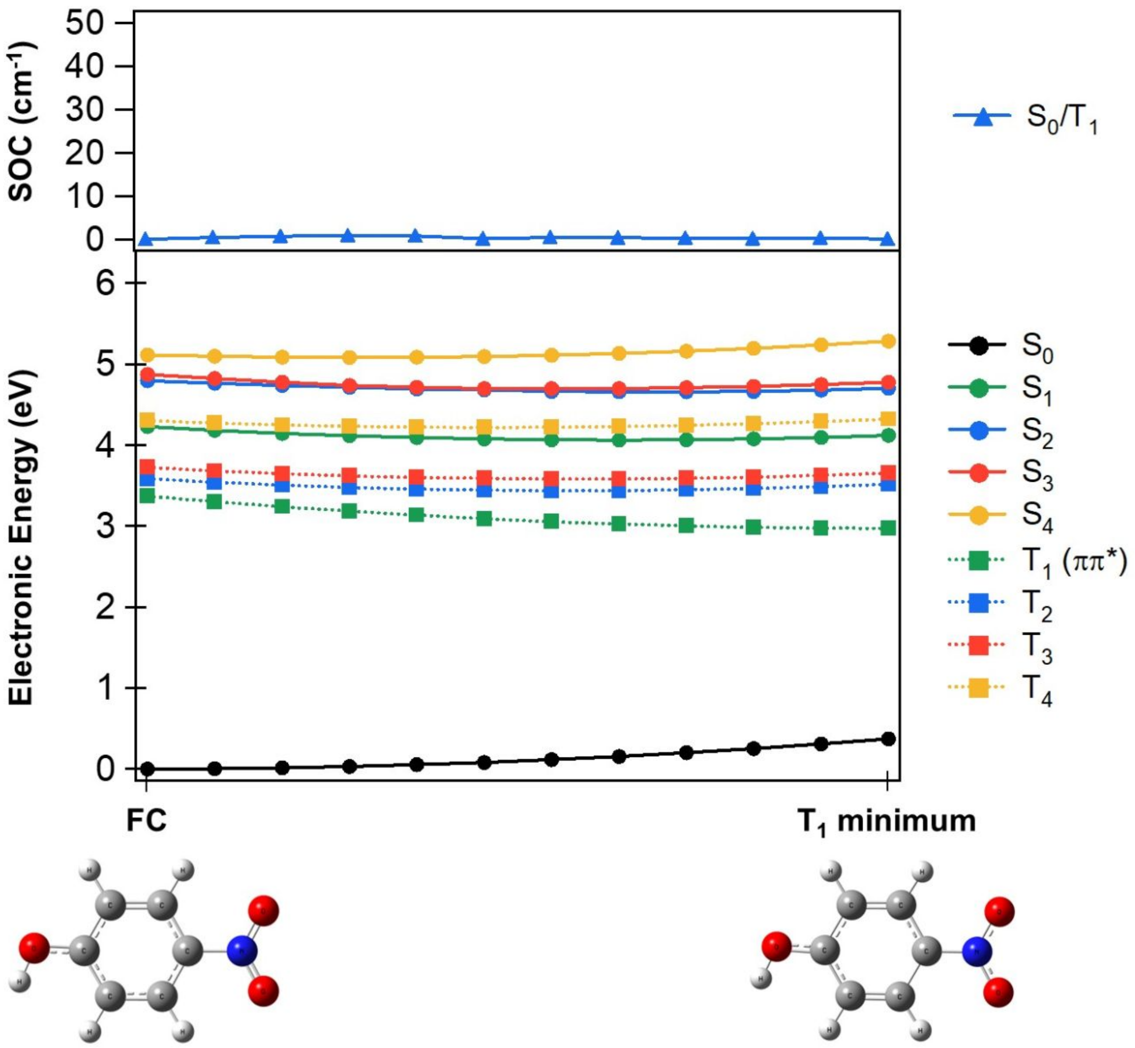

Figure S17: A geodesic interpolation in internal coordinates using 10 intermediate steps between geometries of the ground state minimum (left hand side geometry) and T_1 minimum (right hand side geometry). Energies of the ground and excited states relative to the S_0 minimum (lower panel) and the S_0-T_1 spin orbit coupling (SOC) at each geometry (upper panel) are calculated using (LR-TD)DFT/TDA/ωB97-X-D3/ZORA-def2-SV(P) with a non-equilibrium CPCM.

Table S6: Kinetic time constants of the ground (S_0) state p-nitrophenolate band (1584 cm^{-1}) extracted from TRIR measurements for p-NP in solutions of different pD.			
pD	τ_{PT} **(growth)**	τ_{Diff}	τ
**3**	4.4 ± 0.1 ns	101.4 ± 1.4 ns	
**5**	4.3 ± 0.1 ns	152.7 ± 3.5 ns	
**5, 1M CsCl**	4.8 ± 0.1 ns	131.4 ± 13.1 ns	836 ± 58 ns
(5, CsCl) represents 1M CsCl added to pD 5 solution.			

Section S5: Experimental Methodology

S5.1: TA set-up at the University of Bristol (UoB)

TA measurements at UoB were conducted using a combination of an ultrafast laser oscillator (Coherent Vitara-S) and amplifier (Legend Elite HE+), producing 800-nm laser pulses with a duration of 55 fs, a repetition rate of 1 kHz, and a total power of 4.3 W. A beam splitter separated 2% of the output power to generate a white light continuum (WLC) probe beam spanning 350 – 700 nm for TA experiments. The remaining 98% was divided by a 50:50 beam splitter to pump two optical parametric amplifiers (OPAs), one of which was employed to generate 320 nm and 400 nm pump pulses for aqueous solutions of p-nitrophenol (p-NP) and p-nitrophenolate, respectively.

To attenuate the energy of the pump light to approximately 450 nJ per pulse, a half-wave plate was utilized. The polarizer was set at the magic angle (54.7°) relative to the polarization of the probe pulses to eliminate anisotropy effects. A retroreflector mounted on a movable stage regulated the time delays between the pump pulses and the WLC probe pulses, up to a maximum of 1.2 ns. A 500-Hz mechanical chopper blocked every second pulse of the pump beam for a comparison of pump-on and pump-off measurements to generate TA spectra.

The WLC and pump beams were focused into the sample using an *f* = 75-mm off-axis concave aluminum mirror and an *f* = 200-mm CaF_2 lens, respectively. In previous measurements with our experimental setup, the diameter of the pump beam at the sample was determined to be 250 μm using a Thorlabs BP209-Vis/M beam profiler.[7] After passing through the sample, the pump beam was blocked, and the WLC was re-collimated before passing through a 2-mm cuvette containing a copper sulfate solution (≥ 99.9%, VWR Chemicals) to remove any residual 800-nm light. The transmitted WLC was then focused into an Andor spectrometer (Shamrock 163) fitted with a 1024-element photodiode array (Entwicklungsbüro Stresing) to

obtain a TA spectrum. Holmium oxide glass was used to calibrate the pixel-to-wavelength conversion for the transient spectra. TA spectra were corrected for frequency chirp in the WLC using the KOALA program.[8] The instrument response function was determined to be 110 fs.

S5.2: TA set-up at University College London (UCL)

TA spectra were acquired at UCL using a commercial transient absorption spectrometer (Ultrafast Systems Helios Fire). Femtosecond laser pulses were generated by a regenerative amplifier, seeded by a Ti:Sapphire oscillator (Coherent Astrella-HE-USP). The 320-nm pump wavelength was produced using an optical parametric amplifier (OPA, Coherent OPerA Solo), with the pump energy set at approximately 340 nJ at the sample. The WLC probe beam was generated by focusing the 800-nm fundamental beam into a calcium fluoride plate, providing a probing range of 360-650 nm. The relative polarizations of the pump and probe beams were adjusted to the magic angle of 54.7°. The instrument response function was determined to be around 300 fs through fitting to solvent-only spectra.

S5.3: TA set-up at the Rutherford Appleton Laboratory (RAL)

Time-resolved UV-VIS-NIR absorption experiments at the Rutherford Appleton Laboratory were performed using the Central Laser Facility's synchronized dual amplifier-based multiple probe spectroscopy setup in the LIFEtime Facility.[9] Wavelength tuneable excitation pulses at 1-kHz repetition rate were generated from an OPA pumped by an Yb-based laser amplifier (see below) for excitation of the samples. 100 kHz repetition rate VIS-NIR supercontinuum probe pulses spanning 500 – 875 nm were generated from a second, synchronized Yb-based laser amplifier by focusing a small fraction of the fundamental output (1030 nm) into a 4-mm sapphire window. UV-VIS WLC probe pulses spanning 350 – 460 nm were produced by focusing a portion of the second harmonic of the laser (515 nm) into a 4-mm CaF_2 window. The timings of the pump and probe pulses at the sample were controlled by optical delays and oscillator seed pulse picking for 300 fs – 10 μs, with subsequent multiple probing at the 100-kHz repetition rate of the probe pulses used to acquire longer timescale measurements.[9] The probe spot size at the sample (FWHM) was 30 μm, and the pump spot size was 100 μm. The pump laser polarization was tuned to the magic angle with respect to the probe polarization using an achromatic half-wave plate. Transmitted probe pulses were detected on a Si-array detector (Teledyne Octoplus) at 100 kHz. Absorption difference spectra were obtained from the pump-on and pump-off measurements as described before.[9]

S5.4: TRIR set-up at RAL

All TRIR data were acquired using the LIFEtime facility at RAL.[9, 10] In the LIFEtime laser system, the 1030-nm output of a laser oscillator (80 MHz) seeded two Yb:KGW amplifiers (Light Conversion, Pharos, 15 W, 100 kHz, 260-fs output and Light Conversion, Pharos SP,

6W, 100 KHz, 180 fs). The Pharos amplifier pumped an optical parametric amplifier (OPA; Light Conversion, Orpheus HP) to generate the UV-Vis pump excitation wavelength, and the Pharos SP amplifier pumped two OPAs (Light Conversion, Orpheus ONE) from which a pair of independently tunable mid infra-red (IR) probe beams were obtained by difference frequency generation. The two probe beams were focused by an *f* = 7.5-cm gold parabolic mirror to achieve identical spot sizes. The repetition rate of the UV pump pulses was reduced to 1 kHz by pulse picking to implement multiple-probe-pulse measurements following each excitation pulse. The timing between the UV-Vis pump pulses and the multiple IR probe pulses was controlled using a combination of a 0 – 16 ns optical delay stage and electronic pulse selection, giving efficient measurement of time delays from <1 ps – 8 μs. The pump pulse energy for the 320 and 400-nm wavelengths was approximately 400 nJ at the sample.

For TRIR measurements, the UV pump and two IR probe beams were spatially overlapped at the sample and the transmitted IR beams were separately dispersed by gratings onto a pair of 128-element MCT array detectors (InfraRed Associates, Inc.) to generate TRIR spectra. The probe IR region was selected in the range 1400 - 1700 cm^{-1}, with the central wavenumbers of the two probe pulses detuned to cover separate but overlapping spectral windows and each pulse spanning approximately 200 cm^{-1}. Reference spectra of polystyrene were used to calibrate the pixel-to-wavenumber conversion of the TRIR data after acquisition.

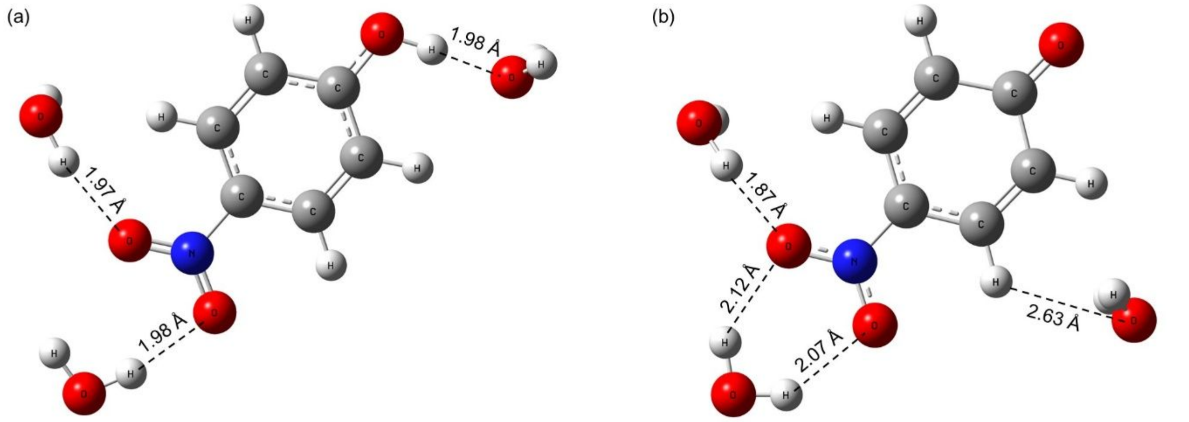

Figure S18: Optimized structures of microsolvated (a) neutral p-NP and (b) p-nitrophenolate anion computed using the DFT/ωB97-X-D3/def2-SV(P) and DFT/ωB97-X-D3/def2-TZVP levels of theory in a PCM cavity, respectively. In each case, microsolvation is with three explicit water molecules.

References

(1) Lau, N.; Ghosh, D.; Bourne-Worster, S.; Kumar, R.; Whitaker, W.; Heitland, J.; Davies, J. A.; Clark, I. P.; Karras, G.; Greetham, G. M.; et al. Unraveling the Ultrafast Photochemical Dynamics of Nitrobenzene in Aqueous Solution. *J. Am. Chem. Soc.* **2024**, *146* (15), 10407-10417. DOI: https://doi.org/10.1021/jacs.3c13826.

(2) Pavitt, A. S.; Bylaska, E. J.; Tratnyek, P. G. Oxidation potentials of phenols and anilines: correlation analysis of electrochemical and theoretical values. *Environ. Sci. Proc. Imp.* **2017**, *19* (3), 339-349, 10.1039/C6EM00694A. DOI: 10.1039/C6EM00694A.

(3) Trasatti, S. The absolute electrode potential: an explanatory note (Recommendations 1986). *Pure Appl. Chem.* **1986**, *58* (7), 955-966. DOI: doi:10.1351/pac198658070955.

(4) Zhan, C.-G.; Dixon, D. A. The Nature and Absolute Hydration Free Energy of the Solvated Electron in Water. *J. Phys. Chem. B* **2003**, *107* (18), 4403-4417. DOI: 10.1021/jp022326v.

(5) Oliver, T. A. A.; Zhang, Y.; Roy, A.; Ashfold, M. N. R.; Bradforth, S. E. Exploring Autoionization and Photoinduced Proton-Coupled Electron Transfer Pathways of Phenol in Aqueous Solution. *J. Phys. Chem. Lett.* **2015**, *6* (20), 4159-4164. DOI: 10.1021/acs.jpclett.5b01861.

(6) Michenfelder, N. C.; Ernst, H. A.; Schweigert, C.; Olzmann, M.; Unterreiner, A. N. Ultrafast stimulated emission of nitrophenolates in organic and aqueous solutions. *Phys. Chem. Chem. Phys.* **2018**, *20* (16), 10713-10720, 10.1039/C7CP07774B. DOI: 10.1039/C7CP07774B.

(7) Roberts, G. M.; Marroux, H. J. B.; Grubb, M. P.; Ashfold, M. N. R.; Orr-Ewing, A. J. On the Participation of Photoinduced N-H Bond Fission in Aqueous Adenine at 266 and 220 nm: A Combined Ultrafast Transient Electronic and Vibrational Absorption Spectroscopy Study. *J. Phys. Chem. A* **2014**, *118* (47), 11211-11225. DOI: 10.1021/jp508501w.

(8) Grubb, M. P.; Orr-Ewing, A. J.; Ashfold, M. N. R. KOALA: A program for the processing and decomposition of transient spectra. *Rev. Sci. Instrumen.* **2014**, *85* (6). DOI: 10.1063/1.4884516.

(9) Greetham, G. M.; Donaldson, P. M.; Nation, C.; Sazanovich, I. V.; Clark, I. P.; Shaw, D. J.; Parker, A. W.; Towrie, M. A 100 kHz Time-Resolved Multiple-Probe Femtosecond to Second Infrared Absorption Spectrometer. *Appl. Spectrosc.* **2016**, *70* (4), 645-653. DOI: 10.1177/0003702816631302.

(10) Koyama, D.; Donaldson, P. M.; Orr-Ewing, A. J. Femtosecond to microsecond observation of the photochemical reaction of 1,2-di(quinolin-2-yl)disulfide with methyl methacrylate. *Phys. Chem. Chem. Phys.* **2017**, *19* (20), 12981-12991, 10.1039/C7CP01784G. DOI: 10.1039/C7CP01784G.
(11) El-Sayed, M. A. Spin—Orbit Coupling and the Radiationless Processes in Nitrogen Heterocyclics. *J. Chem. Phys.* **1963**, *38* (12), 2834-2838. DOI: 10.1063/1.1733610.
(12) Lower, S. K.; El-Sayed, M. A. The Triplet State and Molecular Electronic Processes in Organic Molecules. *Chem. Rev.* **1966**, *66* (2), 199-241. DOI: 10.1021/cr60240a004.
